# Metastatic phenotype and immunosuppressive tumour microenvironment in pancreatic ductal adenocarcinoma: Key role of the urokinase plasminogen activator (PLAU)

**DOI:** 10.3389/fimmu.2022.1060957

**Published:** 2022-12-14

**Authors:** S. M. Zahid Hosen, Md. Nazim Uddin, Zhihong Xu, Benjamin J. Buckley, Chamini Perera, Tony C. Y. Pang, Alpha Raj Mekapogu, Mohammad Ali Moni, Faiyaz Notta, Steven Gallinger, Ron Pirola, Jeremy Wilson, Marie Ranson, David Goldstein, Minoti Apte

**Affiliations:** ^1^ Pancreatic Research Group, SWS Clinical Campus, School of Clinical Medicine, Faculty of Medicine and Health, UNSW Sydney, Sydney, NSW, Australia; ^2^ Ingham Institute for Applied Medical Research, Liverpool, NSW, Australia; ^3^ Institute of Food Science and Technology, Bangladesh Council of Scientific and Industrial Research (BCSIR), Dhaka, Bangladesh; ^4^ Molecular Horizons and School of Chemistry & Molecular Bioscience, Faculty of Science, Medicine and Health, University of Wollongong, Wollongong, NSW, Australia; ^5^ Illawarra Health and Medical Research Institute, Wollongong, NSW, Australia; ^6^ Westmead Clinical School, Faculty of Medicine and Health, University of Sydney, The University of Sydney, Sydney, NSW, Australia; ^7^ School of Health and Rehabilitation Sciences, Faculty of Health and Behavioural Sciences, The University of Queensland, St Lucia, QLD, Australia; ^8^ PanCuRx Translational Research Initiative, Ontario Institute for Cancer Research, Toronto, ON, Canada; ^9^ Prince of Wales Clinical School, University of New South Wales, Sydney, NSW, Australia; ^10^ Department of Medical Oncology, Prince of Wales Hospital, Randwick, NSW, Australia

**Keywords:** *PLAU*, pancreatic stellate cells, proliferation, EMT, stemness, ECM degradation, immune suppression and basal subtype type of PDAC

## Abstract

**Background:**

Previous studies have revealed the role of dysregulated urokinase plasminogen activator (encoded by *PLAU*) expression and activity in several pathways associated with cancer progression. However, systematic investigation into the association of *PLAU* expression with factors that modulate PDAC (pancreatic ductal adenocarcinoma) progression is lacking, such as those affecting stromal (pancreatic stellate cell, PSC)-cancer cell interactions, tumour immunity, PDAC subtypes and clinical outcomes from potential *PLAU* inhibition.

**Methods:**

This study used an integrated bioinformatics approach to identify prognostic markers correlated with PLAU expression using different transcriptomics, proteomics, and clinical data sets. We then determined the association of dysregulated *PLAU* and correlated signatures with oncogenic pathways, metastatic phenotypes, stroma, immunosuppressive tumour microenvironment (TME) and clinical outcome. Finally, using an *in vivo* orthotopic model of pancreatic cancer, we confirmed the predicted effect of inhibiting PLAU on tumour growth and metastasis.

**Results:**

Our analyses revealed that *PLAU* upregulation is not only associated with numerous other prognostic markers but also associated with the activation of various oncogenic signalling pathways, aggressive phenotypes relevant to PDAC growth and metastasis, such as proliferation, epithelial-mesenchymal transition (EMT), stemness, hypoxia, extracellular cell matrix (ECM) degradation, upregulation of stromal signatures, and immune suppression in the tumour microenvironment (TME). Moreover, the upregulation of *PLAU* was directly connected with signalling pathways known to mediate PSC-cancer cell interactions. Furthermore, *PLAU* upregulation was associated with the aggressive basal/squamous phenotype of PDAC and significantly reduced overall survival, indicating that this subset of patients may benefit from therapeutic interventions to inhibit *PLAU* activity. Our studies with a clinically relevant orthotopic pancreatic model showed that even short-term PLAU inhibition is sufficient to significantly halt tumour growth and, importantly, eliminate visible metastasis.

**Conclusion:**

Elevated *PLAU* correlates with increased aggressive phenotypes, stromal score, and immune suppression in PDAC. *PLAU* upregulation is also closely associated with the basal subtype type of PDAC; patients with this subtype are at high risk of mortality from the disease and may benefit from therapeutic targeting of *PLAU*.

## 1 Introduction

Pancreatic ductal adenocarcinoma (PDAC), the most common subtype of pancreatic cancer (PC), is currently the seventh leading cause of cancer-associated death (1) and has a notoriously dismal prognosis. The incidence of PDAC continues to increase, and it is projected to become the second most common cause of cancer-linked death by 2030 (2). Current treatments have limited impact. The mean overall survival of the current standard treatment of FOLFIRINOX is 12.5 months, and that of Gemcitabine plus Abraxane, 10.3 months, P = 0.05 (3). Immunotherapies, individually or in combination with chemoradiotherapy or targeted therapy, have not made much progress in PDAC (4–7), reflecting an urgent need to identify new biologically driven targets to limit PDAC progression, particularly metastasis, the primary driver of mortality in this disease.

PDAC is no longer considered one disease at the molecular level, with many different molecular subtypes and subtype-specific treatment responses in PDAC (4–6). The two major transcriptomic-based subtypes, which have been confirmed across multiple investigations, are the classical/pancreatic progenitor subtype and the quasi-mesenchymal/basal-like/squamous subtype (4, 5, 8). The basal subtype is over-represented amongst metastatic PDAC tumours, and it is distinguished by ECM-rich activated stroma, the upregulation of expression of laminins and keratins and enriched for genes associated with epithelial-mesenchymal transition (EMT) and TGF-β signalling (9). On the other hand, the classical PDAC signature is characterised by upregulation of a wide range of transcription factors, GATA4, GATA6, NKX2-2 and HNF1A, associated with pancreatic lineage differentiation (4–8). Clinicopathologically, basal-type tumours are poorly differentiated and correlate with a worse prognosis (median OS 10–19.2 months and DFS 4.6–10.9); these tumours are chemoresistant but may have a better response to adjuvant therapy (4–6, 9–13). In contrast, classical type tumours are well-differentiated and are correlated with an overall better prognosis (median OS 19–43.1 months and DFS 13.5–20.6) (4, 6, 8, 10, 14–17).

Histologically, PDAC is well known to be characterised by a prominent stromal reaction comprising non-cellular elements like collagen, fibronectin, glycoproteins, proteoglycans, hyaluronic acid, cytokines, growth factors, and serine protein acidic and rich in cysteine (SPARC), as well as a wide range of cell types including neural, endothelial, immune & pancreatic stellate cells (PSCs). Pancreatic stellate cells (PSCs) are responsible for producing this excessive collagenous stroma in PDAC (18–20). Reciprocal interactions between activated pancreatic stellate and PDAC cells facilitate PDAC development and progression. One of the key pathways that may mediate cancer-stromal interactions is the hepatocyte growth factor (HGF) and its receptor c-MET pathway. Hyperactivity of HGF/c-MET signalling is considered a hallmark of cancer. Further, the serine protease urokinase plasminogen activator (uPA, encoded by *PLAU*) activates pro-HGF (secreted by pancreatic stellate cells) to active HGF, which binds to the c-MET receptor on cancer cells, activating several downstream signalling molecules. In addition, HGF binding to the c-MET receptor induces *PLAU* production by pancreatic and other cancer cells. The increased uPA level further activates pro-HGF, resulting in a feed-forward activation loop to promote cancer progression (21–23).

In normal cells (24–27), *PLAU* expression is very low and tightly controlled (7, 23, 28). However, *PLAU* and subsequently uPA expression is increased several-fold in tumour cells (23, 29–31), which results in catalytic conversion of inactive plasminogen to plasmin. Plasmin degrades extracellular matrix directly or indirectly *via* activation of precursor forms of matrix-degrading enzymes (matrix metalloproteinases) (32). Furthermore, in cancer cells, direct interaction of uPA with its receptor uPAR (encoded by PLAUR) facilitates the activation of multiple intracellular cell-signalling pathways, which regulate proliferation, migration, invasion, epithelial-mesenchymal transition, stem cell-like properties, release from states of dormancy, cell survival, chemoresistance, angiogenesis and vasculogenic mimicry (7, 24–27, 33–41) in cancer. All of which suggests a role as a master regulator in cancer development and progression. Indeed, upregulation of *PLAU* is associated with poor prognosis in several different cancers (33, 42). One study analysed 8 PDAC versus normal tissue gene expression profiles retrieved from the GEO database and found *PLAU* and *PLAU*R to be one of 10 hub genes significantly associated with PDAC pathogenesis (43).

This is the first study to delineate the role of the *PLAU* by integrated publically available transcriptomic, proteomics, and clinical data to 1) further elucidate the mechanisms underlying *PLAU*-related PDAC growth and progression, 2) use this data to undertake analyses of prognostic outcomes (overall survival) and assessment of relationship with clinical attributes, 3) identify the most ‘at risk’ group based on *PLAU* expression and4) preclinically assess selective uPA inhibition on pancreatic cancer growth and metastasis. To the best of our knowledge, this is the first integrated–omics analysis of the expression of these key components of the uPA system in PDAC.

## 2 Materials and methods

**Figure 1:**
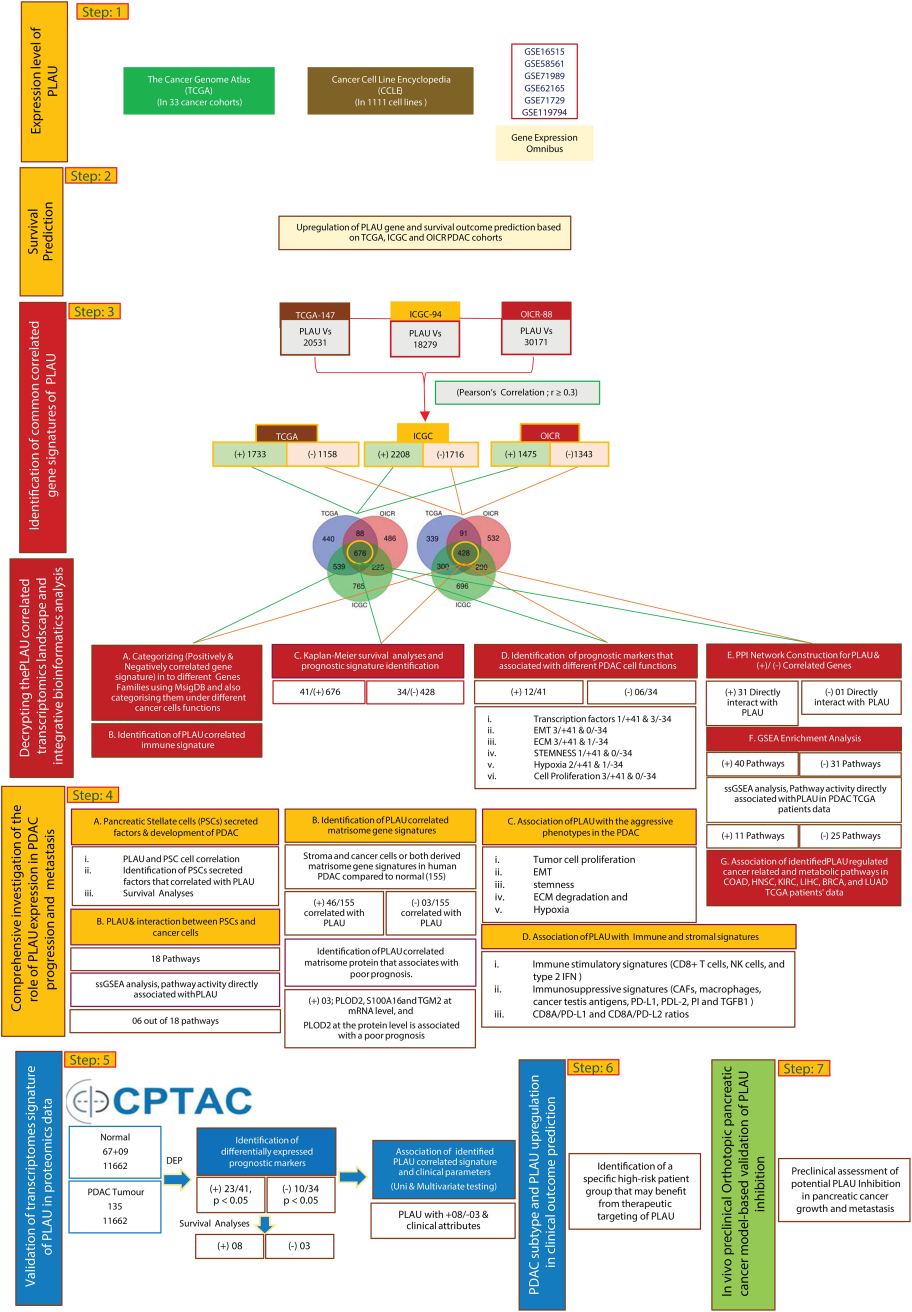
An integrative clinical bioinformatics workflow to decipher the role of *PLAU* in PDAC growth and progression, clinical outcome prediction and *in vivo* preclinical method-based validation of *PLAU* inhibition effects in tumour growth and metastasis.

This study was implemented according to the analytical approach shown in **Figure 1**. The main steps involved in this task were step 1) identification of differentially expressed *PLAU* mRNA in i) 33 different cancer cohorts in the TCGA database, ii) different cancer cell lines from CCLE and iii) different GEO datasets. Step 2) Kaplan-Meier survival analyses of *PLAU* in PDAC-specific TCGA, ICGC and OICR patient cohorts. Step 3) Identifying other gene signatures correlated with *PLAU* from TCGA, ICGC and OICR patients cohorts. These gene signatures were mainly related to cancer cell functions, immunity and prognosis. A PPI network was constructed based on the gene signatures, and relevant subcellular pathways were identified. Step 4) Assessing the correlation of *PLAU* expression with pathways responsible for PSC-PDAC cell interactions. Step 5) Validation of transcriptome-based prognostic signatures using CPTAC proteomics data and assessing the relationship with clinical attributes. Step 6) Stratifying patient groups according to PLAU protein expression and survival and identifying the most ‘at risk’ group. Step 7) Further validation of the effect of PLAU inhibition on PDAC tumour growth and metastasis using *in vivo* pancreatic orthotopic model.

### 2.1 Datasets

We used the GEPIA (Gene Expression Profiling Interactive Analysis) TCGA dataset (http://gepia.cancer-pku.cn/) for comparing the differential mRNA expression of *PLAU* between cancer and normal samples. The Cancer Cell Line Encyclopedia (CCLE) (https://www.broadinstitute.org/ccle) mRNA expression data was used to identify distinctively upregulated *PLAU* in pancreatic cancer cell lines (44, 45). Next, we used different microarray data sets, including GSE16515 (46), GSE58561 (47), GSE71989 (48), GSE62165 (12), GSE71729 (6), and RNAseq GSE119794 (49) from the NCBI-GEO database.

Messenger RNA expression data and associated clinicopathological data were used from The Cancer Genome Atlas (TCGA, http://cancergenome.nih.gov/) and the International Cancer Genome Consortium (ICGC, https://icgc.org/). In particular, normalised gene expression of NGS was downloaded from the cBioPortal, (TCGA, Firehose https://www.cbioportal.org/) (50) on 1^st^ July 2021. For the ICGC-Pancreatic Cancer - Australia (ICGC-PACA-AU) cohort, data were obtained from the **Supplementary Material** of the corresponding publication (4). In addition, we also used the Ontario Institute for Cancer Research (OICR) PDAC cohort (EGAS00001002543) for gene expression and clinical data through a data access agreement. Likewise, the proteomic and accompanying clinicopathological data from the proteogenomic characterisation of the PDAC study (6) was acquired *via* the Clinical Proteomic Tumor Analysis Consortium (CPTAC, https://cptac-data-portal.georgetown.edu/). Only PDAC cases with matched RNAseq/protein expression and clinical data were included in the analysis for all the cohorts.

### 2.2 Differential expression analysis

Differential expression analysis was performed using GEO2R (http://www.ncbi.nlm.nih.gov/geo/geo2r) and R packages limma from the Bioconductor project (http://www.bioconductor.org/). The thresholds of P-value < 0.05 and |FC| (fold change) > one was set to determine the significant level.

### 2.3 Identification of correlated gene signature

We used Pearson’s correlation test to identify gene-gene correlation because the expression data is normally distributed. However, we employed Spearman’s correlation test between the mRNA expression level of *PLAU* and the ssGSEA score of selected pathways because the data is not normally distributed. The threshold of our correlation analysis was set at greater than 0.30, and FDR ≤0.05. A false discovery rate (FDR) calculated by the Benjamini and Hochberg method (51) was used to adjust for multiple tests.

By comparing annotated gene sets from the Molecular Signatures Database (MSigDB) (52), and using the online tool “Calculate and draw custom Venn diagrams” (http://bioinformatics.psb.ugent.be/webtools/Venn/) we identified common tumour suppressors, oncogenes, translocated cancer genes, transcription factors, cytokines and growth factors, protein kinases, homeodomain proteins, and cell differentiation markers among positive and negatively correlated gene signatures of *PLAU* identified from three PDAC cohorts.

### 2.4 Gene-set enrichment analysis

We performed gene-set enrichment analysis of the *PLAU*-correlated genes using GSEA (53) with a false discovery rate threshold, FDR < 0.05. The Kyoto Encyclopedia of Genes and Genomes (KEGG) pathways that were significantly associated with the positive and the negatively *PLAU*-correlated genes were also identified (FDR < 0.05).

### 2.5 Functional analysis

We constructed protein-protein interaction (PPI) networks of the *PLAU*-correlated genes using the STRING (version v11 (54)) and visualised the PPI networks by utilising the Cytoscape 3.9.1 software (55). The rank of genes was identified by the Cytoscape plugin cytoHubba (56). Hub nodes were identified using a threshold of medium interaction score ≥0.40, and we selected the degree of interaction ≥25 for identifying the most closely interacting genes in the PPI.

### 2.6 Survival analysis

We used the clinical data of TCGA, ICGC, OICR and CPTAC PDAC cohorts for survival analysis. We compared the overall survival (OS) between PDAC patients classified based on gene expression levels (high expression levels >mean > low expression levels). Kaplan-Meier survival curves were used to show the survival time differences, and the log-rank test was utilised to evaluate the significance of survival time differences between both groups. We used the R package “survival” to perform survival analysis (57), and the function “coxph” in the R package “survival” was used for the u*nivariate* and *multivariable Cox regression analyses (* 57).

### 2.7 Evaluation of immune scores, stromal scores, and tumour purity in stromal content

We utilised the “ESTIMATE” R package to calculate an immune score representing the enrichment levels of immune cells and a stromal score representing the content of stromal cells (58) in the TCGA-PDAC cohort. We compared immune and stromal scores between the patients with high expression of *PLAU* and low expression of the *PLAU* group in PDAC (high expression levels >mean > low expression levels). We considered the Wilcoxon sum rank test (P-value ≤0.05) to identify significant differences between both groups.

### 2.8 Associations of the expression levels of *PLAU* with immune signature, pathway activity, and tumour phenotypes in PDAC

We first identified the *PLAU* correlated cell function and immune gene signatures. Then we used the single-sample gene-set enrichment analysis (ssGSEA) to quantify the enrichment scores of immune and stromal signatures in tumours based on the expression levels of their marker genes (53). We defined the ratio of immune signatures in a tumour sample as the ratio of the average expression levels of their marker genes. The immune and stromal signatures analysed included B cells, CD8+ T cells, CD4+ regulatory T cells, macrophages, neutrophils, natural killer (NK) cells, tumour-infiltrating-lymphocytes (TILs), regulatory T cells (Tregs), cytolytic activity, T cell activation, T cell exhaustion, T follicular helper cells (Tfh), M2 macrophages, tumour-associated macrophage (TAM), T helper 17 cells, myeloid-derived suppressor cell (MDSC), endothelial cell, and cancer-associated fibroblasts (CAFs). Their marker genes are shown in **Supplementary Table (ST) B**. Moreover, we identified the ssGSEA score of all enriched pathways that directly correlate with *PLAU* and tumour phenotypes (proliferation, EMT, stemness, ECM degradation, and hypoxia). The genes associated with the specific pathways and phenotypes are listed in **STB, ST12**. Finally, we compared immune signatures and phenotypes of PDAC patients with high expression of *PLAU* with those with low expression of the *PLAU.*

### 2.9 *In vitro* and *in vivo* study

#### 2.9.1 Isolation and characterisation of cancer-associated hPSCs

Using the outgrowth method (59), CAhPSCs were isolated from surgically removed pancreatic tissue obtained from cancer patients. The characterisation of CAhPSC yield was then assessed by morphology and immunostaining for the selective GFAP and the activation marker αSMA (60).

#### 2.9.2 Cell culture

AsPC-1 cells (American Type Culture Collection, Manassas, VA) and CAhPSCs were cultured according to the supplier’s instructions and following previously published protocols by our group (61).

#### 2.9.3 *In vivo* orthotopic model of pancreatic cancer

To validate the outcome of *PLAU* (uPA) inhibition on tumour growth and metastasis *in vivo*, we conducted a pilot study using an orthotopic model of pancreatic cancer as previously established in our laboratory (21, 62, 63). Briefly, 6-8 weeks old female athymic nude mice (BALBc nu/nu) were anaesthetised, and an opening was made in the left flank, followed by exteriorisation of the spleen and tail of the pancreas. Then 1 × 10^6^ human PC cells (AsPC-1) plus 1 × 10^6^ cancer-associated human PSCs (CAhPSCs) in 50μL of PBS were implanted into the tail of the pancreas to replicate early cancer development and progression. Mass Spectrophotometry–Based proteome profiling (ST19) confirmed *PLAU* protein expression in both AsPC1 and CAhPSCs. Seven days after cell implantation, mice were randomised (n=5/group) to receive vehicle control (Ctrl), Gemcitabine (G) 75 mg/kg IP biweekly, uPA small molecule enzymatic inhibitor [5,6-disubstituted amiloride analogue, compound BB2-30F (A26) (64)] 3mg/kg (U3) or 10mg/kg (U10) IP daily. BB230F compounds were formulated for IP injection in 50 mM acetate buffer (pH5.5) + 10% DMSO + 1% Kolliphor HS-15 buffer and filtered through 0.22 μm PVDF syringe-driven filters under sterile conditions (64) The total number of vehicle injections was 28 (daily IP injections), allowing us to control maximally for any effects of IP injections per se in our model. Pancreatic tumour growth was monitored by palpation. At the end of 28 days of treatment, tumours were resected, and tumour volume was determined according to the formula (1/2(length × breadth × width) using digital Vernier callipers (Intech Tools, Thomas Town, VIC, Australia). The abdominal cavity, mesentery, spleen, liver, and lungs were examined, and a metastasis score was calculated based on the presence or absence of visible macrometastatic nodules. Haematoxylin and eosin staining was performed to confirm the presence of such nodules. Primary tumour sections were immunostained for E-cadherin, vimentin and ALDH1A1. Tumour volume data are expressed as mean ± SEM. One-way analysis of variance with Tukey’s *post hoc* test was applied. Analyses were performed using GraphPad Prism 9 for Windows 64-bit (GraphPad Software, San Diego, CA, USA).

The animal studies were approved by the University of New South Wales Animal Care and Ethics Committee (Approval Number 18/125B) and accomplished under ARRIVE guidelines.

## 3 Results

### 3.1 *PLAU* is significantly differentially expressed in various cancers

**Figure 2:**
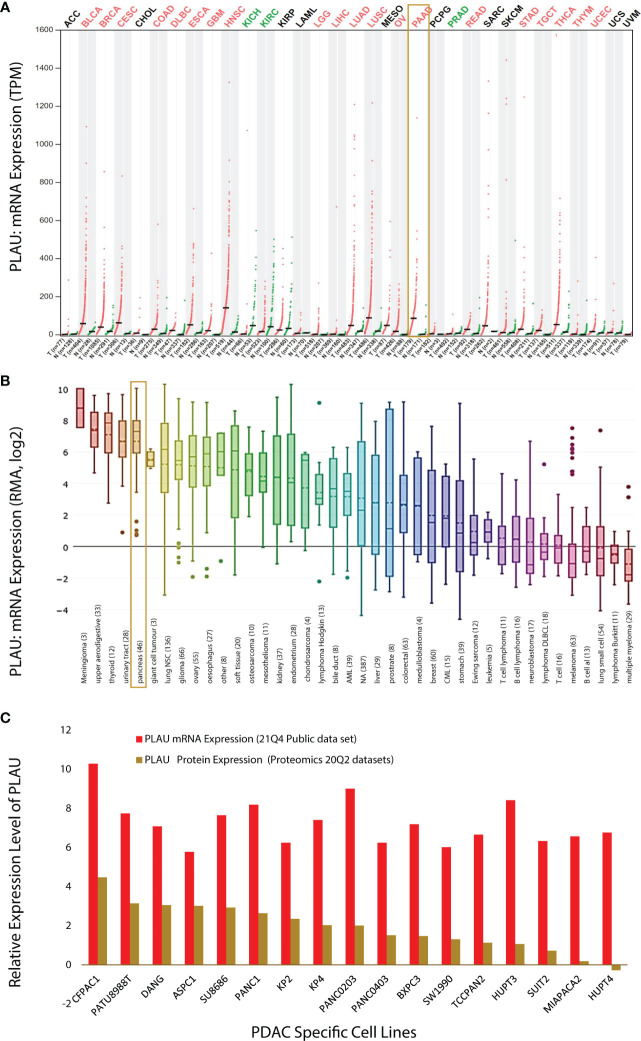
*PLAU* expression in cancers. **(A)** Dot plot depicting *PLAU* gene expression profile across 33 cancer types and paired normal samples (TCGA normal plus GTEx), with each dot representing a distinct tumour or normal sample. The bar height represents the median expression of a certain tumour type or normal tissue. The comparison was performed using GEPIA (Gene Expression Profiling Interactive Analysis). For each TCGA tumour (red), its matched normal and GTEx data (green) are given; T: tumour; N: normal; n: number. Y-axis: transcripts per million log2(TPM + 1). X-axis: number of tumours and normal samples. **(B)** *PLAU* expression across 1111 human cancer cell lines from the Cancer Cell Line Encyclopedia (CCLE). Box plots showing RNA-seq mRNA expression data from CCLE, with the dashed lines within a box representing the mean. Cell lines derived from the same organ/organ system were grouped, and lineages are indicated at the bottom of the graph, with the number of cell lines per organ/organ system in parenthesis. The “pancreas” group includes the 44 pancreatic cancer cell lines listed in (**ST2A**). **(C)** Relative expression level of *PLAU* at mRNA and protein level in 17 PDAC cell lines using the depmap portal.

Using the GEPIA dataset, it was found that *PLAU* mRNA levels are significantly differentially expressed (compared to relevant normal tissue) in 23 of the 33 different types of cancers assessed (**Figure 2A**; Red =tumour and Green= normal). In BLCA, BRCA, CESC, COAD, DLBC, ESCA, GBM, HNSC, KICH, KIRC, LGG, LIHC, LUAD, LUSC, OV, PAAD, PRAD, READ, STAD, TGCT, THCA, THYM, and UCEC *PLAU* is significantly upregulated while in KICH, KIRC and PRAD it is downregulated (**Figure 2A**). In particular, in the PAAD cohort of pancreatic cancer, *PLAU* transcripts were 4.876 (p=1.6e-103) fold elevated compared with normal tissue. In support of the above observations, the Cancer Cell Line Encyclopedia (CCLE) dataset revealed that (https://www.broadinstitute.org/ccle) *PLAU* was also differentially expressed in different cancer cell lines (**Figure 2B**), including 44 pancreatic cancer cell lines (from primary and metastatic PDAC tumours (**ST2 A**). Upregulated mRNA and protein expression levels for *PLAU* (relative to normal controls) in 17 PDAC cell lines are depicted in **Figure 2C**.

The above observations related to PDAC were further confirmed by analysis of several GSE microarrays which showed significant fold increases in *PLAU* mRNA in PDAC vs normal controls as detailed in the following: GSE16515 (logFC 2.73, P=2.32E-07); GSE58561 (logFC 4.94, P = 5.35E-06); GSE71989 (logFC 3.29, P =2.56E-06); GSE62165 (logFC 3.31, P = 1.91E-27); GSE71729 (logFC 1.56, P = 1.98E-09), and RNAseq GSE119794 (logFC 1.256, P= 0.003), **ST3**. Taken together, the above findings indicate that *PLAU* is significantly upregulated in different tumours and cancer cell lines. Of particular relevance to this study, pancreatic cancer and cell lines, consistently demonstrate upregulation of *PLAU* gene expression, suggesting that *PLAU* may play driver roles in the development and progression of PDAC.

### 3.2 Upregulated mRNA expression of *PLAU* is associated with poor survival in PDAC patients

**Figure 3:**
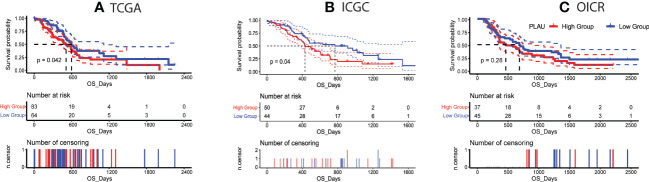
Correlation of *PLAU* gene expression with survival in PDAC. **(A, B)** Kaplan-Meier survival curves show that high *PLAU* expression correlated with significantly poorer overall survival (OS) in the TCGA and ICGC PDAC cohorts (log-rank test, P < 0.05), **(C)** but this was not evident with the OICR-PDAC cohort (log-rank test, P = 0.28).

Given the significant upregulation of *PLAU* in PDAC patients from distinct datasets, we further investigated the association of *PLAU* with clinical outcomes. TCGA data of 147 PDAC patients from 179 PAAD-TCGA cohorts were analysed to reveal that patients in the high *PLAU* mRNA expression group had the poorest outcome (high expression group of *PLAU* > mean expression level of *PLAU* > low expression of *PLAU*) (**Figure 3A**), P=0.042. Similar results were obtained on analysis of the ICGC patient cohort (GSE36924) (**Figure 3B**), P=0.04 (4). With the third patient cohort in our study (OICR; EGAS00001002543) (65), there was a trend for poorer survival in patients with high *PLAU* mRNA expression, but the difference did not achieve statistical significance P= 0.28 (**Figure 3C**). Altogether, these data demonstrate that the upregulation of *PLAU* mRNA expression is an adverse prognostic factor in PDAC.

### 3.3 *PLAU* is significantly correlated with key signal regulatory and tumour immune genes in PDAC

In view of our finding of an association between high *PLAU* gene expression and poor prognosis in PDAC patients, we were interested in analysing other genes that may be correlated with *PLAU* and might influence patient outcomes. Pearson’s correlation coefficient test was used to identify gene-gene correlations for all genes in the expression tables of the TCGA, ICGC, and OICR datasets. A Venn diagram was applied to identify *PLAU*-correlated genes common to all three PDAC datasets (**ST4, ST5** and **Supplementary Figures (SF) 1A, B**). The gene signatures were then categorised into different gene families based on annotated gene sets from Molecular Signatures Database (MSigDB). There were 676 genes positively correlated with the *PLAU* that were common to all three datasets. (**ST 6A, SF 1A**). These included 42 transcription factors (e.g. *FOXC1, HMGA2, RUNX2, SNAI1, SNAI2, TWIST1*, and *WT1*), 16 protein kinases (e.g. *MET, MAPK12*, and *AKT3*), 8 homodomain proteins (including *SIX4, NKX3-2*, and *HLX*)*;*, 27 cell differentiation markers (including *PDL1, CD44, CD70, CDH2*, and *ITGA3)*, 18 oncogenes (e.g. *CDH11, COL1A1*, and *PDGFB)*, 16 translocated cancer genes (*CDH11, CLTCL1, COL1A1, MAF*, and *MAFB)*, one tumour suppressor gene *WT1, and* 38 cytokines and growth factors (including *TGFB2, FGF1, VEGFC, PDGFB, EREG, TGFB1, CCL11, TGFB3, BMP1, IL1,1* and *CCL13)* [Pearson correlation, r>0.3, P> 0.05].

There were 428 genes negatively correlated with the elevated expression levels of *PLAU* that were common to all three datasets (**SF 1B**). These comprised 31 transcription factors (including *CDX2*, *FOXA2, GATA6, HNF1A, HNF4A, PDX1, PPARGC1A*, and *TOX3)*, two cell differentiation markers *(FUT4* and *TNFRSF11A)*, 11 protein kinases (e.g. *ACVR1B, ERBB3, FGFR4, HIPK2, KALRN, PKDCC, SCYL3)*, four translocated cancer genes (including *PRDM16* and *TMPRSS2)*, six oncogenes (including *MYCN, CEBPA*, and *MECOM)*, one tumour suppressor gene (*HNF1A)* and four cytokines and growth factors (including *FAM3B, EDN3, SEMA4G*, and *FAM3D*, **ST 6A**). Several immune-related gene signatures that are positive and negatively associated with *PLAU* were also identified (such as *PDCD1LG2*, *HAVCR2, ANXA1, TNFRSF12A, PLAT, CD276, PTGES, CD44, MMP9, CT45A3, PIWIL2, METTL7A, IL23R, IL17RB, IL22RA1, TNFRSF11A, BLNK*, and *F5*, **ST7**).

Further analysis shows that most of the positively correlated gene signatures of *PLAU* in PDAC regulate cancer cell functions such as cell proliferation, stemness and epithelial-mesenchymal transition, and other factors of importance to cancer biologies such as extracellular matrix degradation, hypoxia, endothelialisation, and metastasis promotion. In contrast, negatively correlated gene signatures were largely uninvolved in cancer cell functions (**ST 6C**, **SF 1E** and **F**).

**Figure 4:**
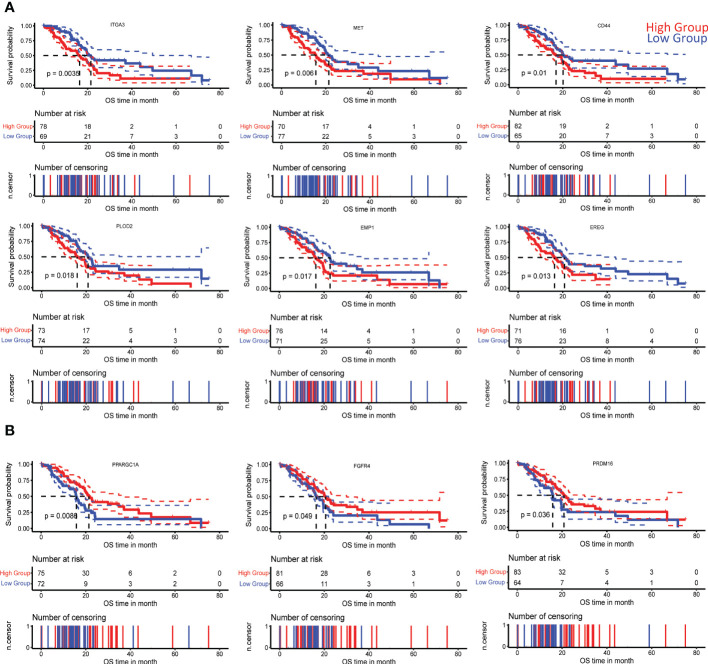
Correlation of *PLAU-associated* genes with survival in PDAC. Kaplan-Meier survival curves show that significantly worse overall survival of PDAC patients in the TCGA cohort is correlated with **(A)** increased expression of ITGA3 (log-rank test, P=0.0035), MET (log-rank test, P = 0.006), CD44 (log-rank test, P= 0.01), PLOD2 (log-rank test, P = 0.018), EMP1 (log-rank test, P = 0.017), EREG (log-rank test, P = 0.013) and **(B)** decreased expression of PPARGC1A (log-rank test, P = 0.0086), FGFR4 (log-rank test, P = 0.049), and PRDM16 (log-rank test, P = 0.036).

A subanalysis of TCGA transcriptomic and clinical data of PDAC patients revealed specific gene signatures (positively and negatively correlated with *PLAU*) associated with poor survival (**ST8**, **SF 1C** and **D**). Among these prognostic genes, we further identified the following positively correlated *MET*, *ITGA3, EREG, PLOD2, EMP1, CD44 HMGA2, TGM2, GAPDH, IL31RA, CGB7, CDH3*, and negatively correlated *PRDM16, PPARGC1A, CAPN6, SPIB, TOX3*, and *FGFR4* associated with the different cancer cell functions listed in **Table 1**, while Kaplan Meier curves signifying their prognostic association are presented in **Figures 4** and **SF 2**.

**Table 1:** *PLAU* correlated genes and their association with cellular functions in PDAC.

Prognostic genes positively correlated with *PLAU*	Factors influencing cancer biology
*HMGA2*	Stemness, oncogene, Transcription Factor and Translocating cancer gene
*TGM2*	Endothelialization, Hypoxia and EMT
*CD44*	ECM degradation, EMT and Cell differential marker
*ITGA3*	ECM degradation and cell differential marker and Metastasis
*MET*	Oncogenes and Protein Kinase
*EREG*	Cell Proliferation, Cytokines and Growth Factor
*GAPDH*	Hypoxia
*PLOD2*	ECM degradation and EMT
*EMP1*	Cell Proliferation
*IL31RA*	Cell Proliferation
*CGB7*	Cytokines and Growth factor
*CDH3*	Metastasis promotion
**Prognostic genes negatively correlated with *PLAU***	**Factors influencing cancer biology**
*PRDM16*	Oncogenes, Transcription Factors, Translocating cancer genes
*PPARGC1A*	Hypoxia, Transcription Factor
*CAPN6*	ECM degradation
*SPIB*	Transcription Factor
*TOX3*	Transcription Factor
*FGFR4*	Cytokines and Growth factor and Protein Kinase

ECM, Extracellular matrix; EMT, Epithelial-mesenchymal transitions.

### 3.4 *PLAU* correlated gene signatures and protein-protein interaction network analysis

The gene analysis described above indicates that upregulated *PLAU* expression is correlated with several key gene signatures that have the potential to influence cancer cell functions and PDAC progression/outcomes. The daunting task is to understand how these positively and negatively *PLAU* correlated genes modulate the PPI network, which can result in dysregulated oncogenic pathways with functional and therapeutic significance. To address this, the 676 positively correlated genes and the 428 negatively correlated genes (common to all three data sets) were entered into the STRING v11 program. 610 of the 676 positively correlated genes and 317 of the 428 negatively correlated genes were involved in the PPI network with PPI enrichment p-value < 1.0e-16 and 3930 edges, and p-value < 1.0e-16 577 edges, respectively. Based on the degree of interactions, some of the top genes within the PPI network were *FN1, GAPDH, COL1A1, CD44, MMP2, COL1A2, MMP9 POSTN, COL5A1, BGN, LOX, COL4A1, MMP14, THBS1*, and *TGFB1* (**ST9A**). Extracting the *PLAU*-centric PPI network from the original extensive network revealed that *PLAU* interacts with 31 of the positively correlated genes (*FN1, MMP2, GAPDH, CD44, MMP9, MMP14, SERPINE1, TIMP2, TGFB1, THBS1, CAV1, MET, MMP13, TIMP3, VEGFC, IGFBP3, CTSB, ITGA5, SNAI1, PLAT, CTSL, CTSD, MMP11, ITGA3, PDGFC, MRC2, PRSS23, SRPX2, KAL1, MFI2*, and *LYPD3*, **SF3** and **ST 9C**), and interestingly, only one negatively correlated gene *ANG* (**ST 9B, D**).

### 3.5 *PLAU* regulates cancer-associated and metabolic pathways in PDAC

**Figure 5:**
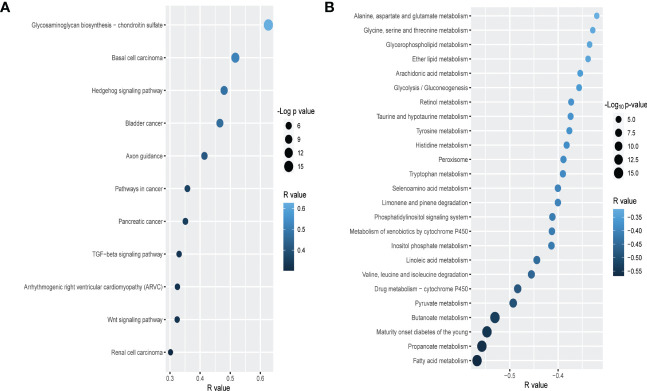
Correlation of *PLAU* upregulation with cancer-associated and metabolic pathways in PDAC. In the TCGA PDAC cohort, upregulation of *PLAU* gene expression is **(A)** directly and positively correlated with cancer-associated pathways and **(B)** negatively correlated with metabolic pathways (FDR<0.05).

To delineate the specific cancer-associated pathways that may be modulated by *PLAU* and its positive/negatively correlated gene signatures, the Functional Class Scoring (FCS) method based on GSEA tool (53) was used (FDR<0.05). Genes that are positively correlated with *PLAU* upregulation were found to be associated with the enrichment of several cancer-associated KEGG pathways (**ST 10A**). In order to assess whether the expression of *PLAU* was directly associated with the activity of these pathways, correlations between the expression levels of *PLAU* (Log2 normalised) and the specific pathway activity (ssGSEA score of the pathway) were analysed for the TCGA-PDAC cohort (Spearman’s correlation test P<0.05). Interestingly, it was found that *PLAU* expression correlated directly with the activity of 11 KEGG pathways, including glycosaminoglycan biosynthesis - chondroitin sulfate, basal cell carcinoma, Hedgehog signalling pathway, axon guidance, pathways in cancer, pancreatic cancer, TGF-beta signalling pathway, arrhythmogenic right ventricular cardiomyopathy (ARVC), wnt signalling pathway, and renal cell carcinoma, FDR<0.01 (**Figure 5A** and **ST 11A**).

Genes that were negatively correlated with *PLAU* upregulation were found to be primarily associated with the enrichment of 31 metabolic pathways, covering the metabolism of specific amino acids, carbohydrates, fatty acids and xenobiotics listed in (**ST 10B**). Interestingly, we discovered that the expression of *PLAU* itself was directly correlated with 25 KEGG pathways (**Figure 5B** and **ST 11B**).

A similar observation of *PLAU* association with cancer-associated and metabolic pathways in various cancers, including COAD, HNSC, KIRC, LIHC, BRCA, and LUAD, was revealed in our further analysis (**SF 4A, B**).

### 3.6 *PLAU* expression is correlated with pancreatic stellate cell -selective markers & pathways in TME of PDAC

**Figure 6:**
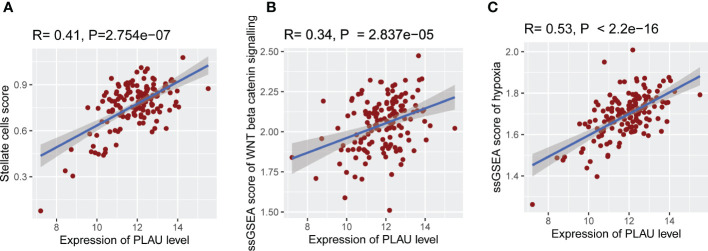
Association of *PLAU* gene expression with abundant activated PSCs and pathways responsible for PSC-PC interactions. In the TCGA PDAC cohort, **u**pregulation of *PLAU* gene expression exhibit a significant positive association with **(A)** abundance of activated PSCs in the TME (R= 0.41, P=2.754e−07), **(B)** WNT beta-Catenin pathway activity (R= 0.34, P=2.837e−05), and **(C)** hypoxia score (R= 0.53, P < 2.2e−16).

As noted earlier, PSCs facilitate the survival and growth of PDAC cells *via* factors that modulate cancer cell proliferation, invasion, migration, metastasis and chemoresistance. In turn, cancer cells activate PSCs *via* the secretion of growth factors and cytokines (*PDGF, VEGF, bFGF, TGF-ß*), resulting in increased PSC proliferation, migration and production of extracellular matrix proteins (66–69). Given this bidirectional interaction between PSCs and cancer cells, we investigated the association of *PLAU* expression with the abundance of activated PSCs. *PLAU* expression was significantly positively correlated (R= 0.41, P=2.754e-07) with the ssGSEA score of PSC-specific markers in the TCGA-PDAC data set (**Figure 6A**). Moreover, a significant moderate correlation was found between *PLAU* and all other secreted markers of activated PSCs (**ST13**), most of which have been shown to play key roles in cancer progression (please see discussion). *PLAU* expression was also correlated with critical pathways known to mediate PSCs-PC interactions (69), including Hedgehog, TGF beta, WNT (**Figure 5A**), WNT beta-Catenin and hypoxia-inducible factor-1 (**Figures 6B, C** **)** signalling pathways.

### 3.7 Identification of prognostically important *PLAU* correlated matrisome gene in human PDAC

**Figure 7:**
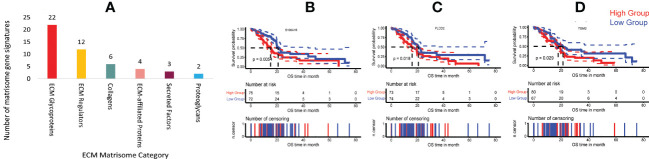
Correlation of *PLAU* expression and matrisome gene signatures in human PDAC. **(A)** Upregulation of *PLAU* gene expression was positively correlated with various PDAC ECM matrisome gene signatures. Kaplan Meier survival curves show that in the TCGA-PDAC cohort, poor survival was associated with **(B)** increased expression of *S100A16*, P = 0.0054, **(C)** *PLOD2*, P = 0.018 and **(D)** *TGM2*, P=0.029.

In the tumour microenvironment, *PLAU* is involved in ECM breakdown through activation of plasminogen to plasmin which activates certain pro-matrix metalloproteinases, facilitating local tumour invasion. Dysregulated ECM proteins also influence tumour progress and patient survival by supporting tumour cell proliferation, angiogenesis, inflammation (22, 28), and metastasis (29, 30). However, the association of *PLAU* with the PDAC-specific matrisome gene (produced by tumour cells and stromal pancreatic stellate cells) has not been assessed in the context of PDAC development and progression. In order to systematically examine the correlations of *PLAU* expression with PDAC-specific ECM gene signatures (from TCGA, ICGC and OICR cohorts), 155 PDAC matrisome gene signatures **(** **ST14** **)** were selected (32 secreted by cancer cells, 87 by stromal cells and 36 from both cancer and stromal cells) (70). 49 ECM gene signatures were found to be correlated with *PLAU*, either positively (33) or negatively (3) (Pearson correlation, *r>0.3; p*< *0.05*). Of the 49 genes, 22 coded for ECM glycoproteins (*EFEMP1, EMILIN1, FBLN2, FBN1, FN1, HMCN1, IGFBP3, LAMA4, LAMC2, LTBP1, LTBP2, MATN2, MFAP2, PCOLCE, POSTN, PXDN, SRPX2, TGFBI, TGM2, THBS1, THBS2* and *TNC*), 12 for ECM regulators (*ADAMTS4, ADAMTSL1, BMP1, CTSB, CTSD, LOX, LOXL1, MMP2, PLOD1, PLOD2, SERPINH1* and *TGM2*), six for collagens (*COL11A1, COL6A1, COL6A2, COL6A3, COL8A1*, and *COL8A2*), four for ECM-affiliated proteins (*ANXA1, ANXA4, LGALS1* and *LGALS4*), three for secreted factors (*S100A16, S100A9*, and *TGFB1*), and two for proteoglycans *BGN* and *VCAN* (**ST14**, **15A** and **Figure 7A**).

Survival analysis of the TCGA-PDAC cohort revealed that secreted factor *S100A16 (*cancer-cell–derived), ECM regulator *PLOD2* (stromal cell-derived) and ECM regulator *TGM2 (*derived from both cancer cells and stromal cells) genes were overexpressed in human PDAC and correlated with short patient survival (log-rank test, P < 0.05), **Figures 7B–D**. In contrast, none of the negatively correlated matrisome gene signatures was associated with patient survival. However, at the protein level (using the CPTAC-PDAC cohort), while PLOD2, S100A16 and TGM2 were all significantly differentially overexpressed in tumours compared to the normal adjacent pancreas (**ST 15B**), only upregulation of PLOD2 (log-rank test, P= 0.05) protein was found to be associated with poor survival (refer to PLOD2).

### 3.8 Upregulation of the *PLAU* gene is correlated with aggressive phenotypes of PDAC

**Figure 8:**
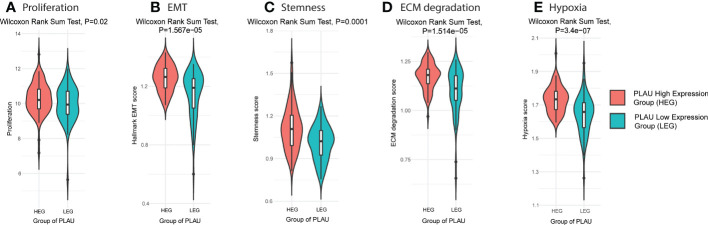
*PLAU* upregulation is associated with aggressive phenotypes of PDAC. Markers of an aggressive phenotype of PDAC were positively correlated with the high *PLAU* expressing group (HEG) compared to the low *PLAU* expression group (LEG), as depicted for **(A)** the tumour cell proliferation and growth index marker, MKI67, Wilcoxon rank-sum test, P= 0.02 **(B)** EMT Wilcoxon rank-sum test, P= 1.567e−05, **(C)** tumour stemness, Wilcoxon Rank Sum Test, P=0.0001, **(D)** ECM degradation, Wilcoxon Rank Sum Test, P= 1.514e−05 **(D, E)** hypoxia, Wilcoxon Rank Sum Test, P= 3.4e−07.

Aggressive PDAC is characterised by increased cancer cell proliferation, EMT, stemness, active ECM and hypoxia. Using the TCGA-PDAC cohort to compare the *PLAU* high expressing group (HEG) vs the *PLAU* low expressing group (LEG), scores for each of the above parameters were found to be elevated (**Figures 8A–E**), and the associated markers significantly correlated (**SF 5A–D**) with high *PLAU* gene expression.

### 3.9 Expression of the *PLAU* gene is associated with an immunosuppressive tumour microenvironment in PDAC

**Figure 9:**
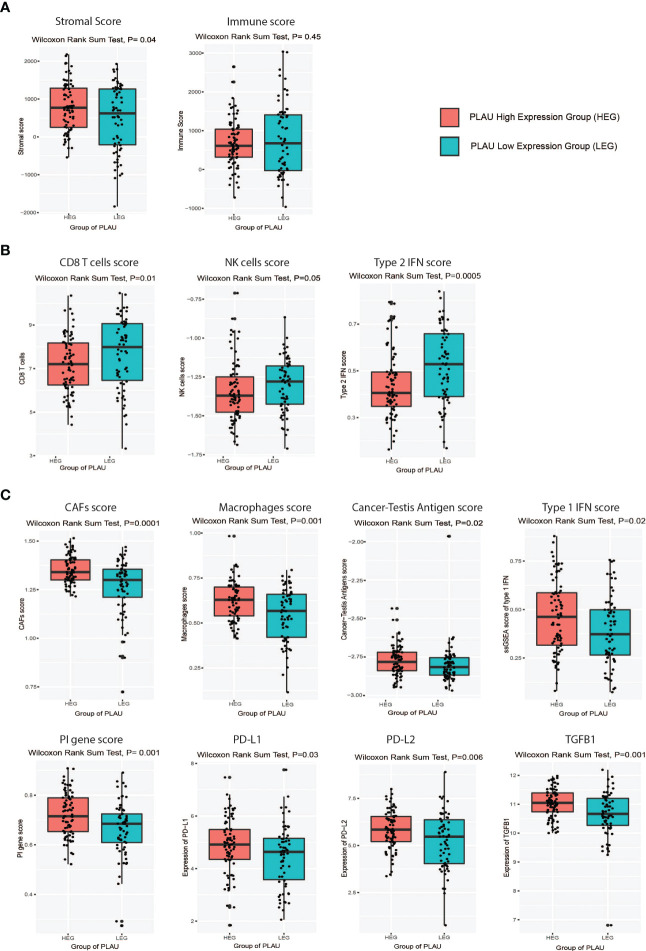
Association of *PLAU* upregulation with an immunosuppressive landscape in the TCGA PDAC cohort. Markers of an immunosuppressive landscape in PDAC were positively correlated with the high *PLAU* expressing group (HEG) compared to the low *PLAU* expression group (LEG), as evidenced by **(A)** a high stromal score (Wilcoxon rank-sum test, P=0.04) and a low immune score (Wilcoxon rank-sum test, P=0.45), **(B)** low scores for immune stimulatory signatures CD8+ T cells (Wilcoxon rank-sum test, P=0.01), NK cells (Wilcoxon rank-sum test, P=0.05), and type 2 IFN (Wilcoxon rank-sum test, P=0.0005), **(C)** high scores for immune inhibitory signatures including CAFs (Wilcoxon rank-sum test, P=0.0001), macrophages (Wilcoxon rank-sum test, P=0.001), cancer-testis antigens (Wilcoxon rank-sum test, P=0.02), Type 1 IFN (Wilcoxon rank-sum test, P=0.02), PI genes (Wilcoxon rank-sum test, P=0.001), PD-L1(Wilcoxon rank-sum test, P=0.03), PDL-2(Wilcoxon rank-sum test, P=0.006), and TGFB1 (Wilcoxon rank-sum test, P=0.001).

**Figure 10:**
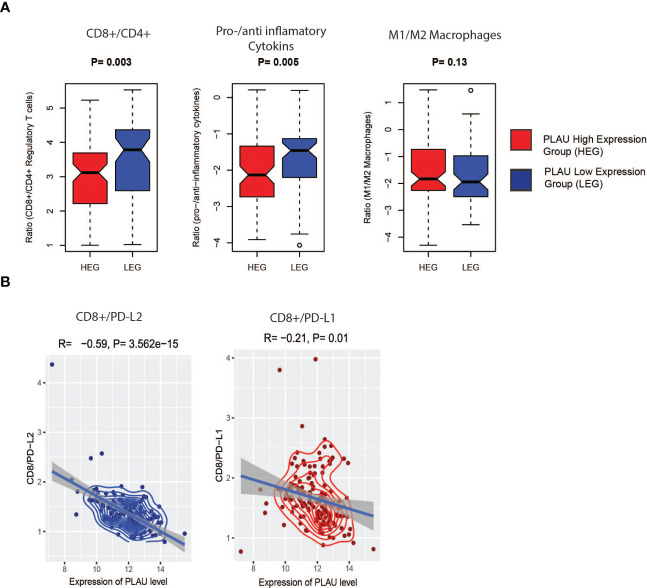
Association of *PLAU* upregulation with the immune ratios in the TCGA PDAC cohort. **(A)** CD8+ T cells/CD4+ T cell (P= 0.003) and pro-/anti-inflammatory cytokines (P= 0.005), significantly lower in the high expression group (HEG) of PLAU and **(B)**, the PLAU expression is negatively correlated with CD8A/PD-L1 (Pearson’s correlation R=-0.59, P= 3.562e-15) and CD8A/PD-L2 (Pearson’s correlation R=-0.21, P= 0.01).

Since the infiltration levels of immune cells are an independent predictor of survival in cancers (58), the differences in various immune and stromal signatures between *PLAU*-high and *PLAU*-low patients in the TCGA-PDAC cohort were examined. Stromal and immune scores were calculated (the content of cells) by applying the ESTIMATE (58) algorithm. The stromal score was significantly higher in the HEG of *PLAU* than in the LEG of *PLAU* (**Figure 9A**, Wilcoxon rank-sum test, p<0.05). In contrast, there was no significant difference in the immune score between the groups. However, the *PLAU*-high group was associated with inhibition of immune stimulatory signatures that included CD8+ T cells, NK cells, and type 2 IFN (**Figure 9B**) and upregulation of immunosuppressive signatures that included CAFs, macrophages, cancer-testis antigens, PI genes, *PD-L1*, *PDL-2*, and *TGFB1* (Wilcoxon rank-sum test, p<0.05) (**Figure 9C**). The ratios of CD8+ T cells/CD4+ T cells and pro-/anti-inflammatory cytokines (as assessed by the ratio of average expression levels (log2-transformed) of their marker genes) were significantly lower in the *PLAU* high group (expression levels > average) (**Figure 10A**, P < 0.05). The pro-inflammatory cytokine genes are immune-stimulatory and include *IFNG*, *IL-1A*, *IL-1B*, and *IL-2*, while the anti-inflammatory cytokine genes *IL-4*, *IL-10*, *IL-11*, and *TGFB1* represent an immune-inhibitory signature. The expression levels of *PLAU* were negatively correlated with CD8A/PD-L1 and CD8A/PD-L2 ratios (Pearson’s correlation test, P < 0.05, **Figure 10B**). Taken together, the above findings indicate that elevated *PLAU* expression has a stronger association with immunosuppressive TME signatures (*PD-L1* and *PD-L2*) than with the anti-tumour immune signature (CD8+ T cells) in the TCGA-PDAC cohort.

### 3.10 *PLAU*-correlated prognostic gene markers are also differentially expressed and associated with poor outcomes in PDAC at the protein level

In order to determine whether the identified prognostic gene signatures (*PLAU* correlated 41 positively and 34 negatively in the TCGA-PDAC cohort) translated to protein or not in the PDAC tumour, we performed a differential expression analysis based on the CPTAC-PDAC cohort. 135 patients’ tumours proteome profile compared with proteins expression data from 67 normal adjacent and nine normal ducts tissues. The results showed that 23 out of 41 positively correlated prognostics markers were differentially upregulated; out of 34 negatively correlated prognostics markers, 16 were differentially downregulated (**ST17**).

**Figure 11:**
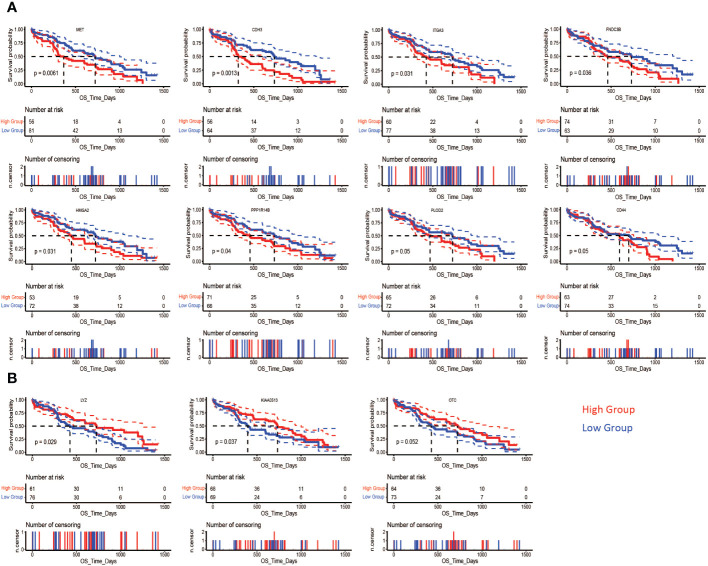
Association of differentially expressed proteins and survival in PDAC. In the CPTAC-PDAC cohort, Kaplan-Meier analysis shows that poor overall survival (OS) was correlated **(A)** high expression of MET (log-rank test, P= 0.0061), CDH3 (log-rank test, P= 0.0013), ITGA3 (log-rank test, P= 0.031), FNDC3B (log-rank test, P= 0.036), HMGA2 (log-rank test, P= 0.031), PPP1R14B (log-rank test, P= 0.04), and PLOD2 (log-rank test, P= 0.05) CD44 (log-rank test, P= 0.05), and **(B)** low expression of LYZ (log-rank test, P= 0.029), KIAA0513 (log-rank test, P= 0.037), and OTC (log-rank test, P= 0.05).

The correlation of the differentially expressed protein signatures noted above with overall patient survival was also assessed in the CPTAC-PDAC cohort. Upregulated expression of CD44, CDH3, FNDC3B, HMGA2, ITGA3, MET, PPP1R14B, and PLOD2 and downregulation of KIAA0513, OTC, and LYZ were associated with poor survival (**Figures 11A, B** **)**. Representative immunohistochemistry images from the human proteome atlas further confirmed the level of expression of the above proteins in PDAC tissues (71) (**SF6**).

### 3.11 Univariate and multivariate cox regression analysis of *PLAU* correlated (survival-related) proteins and different clinicopathological factors

**Figure 12:**
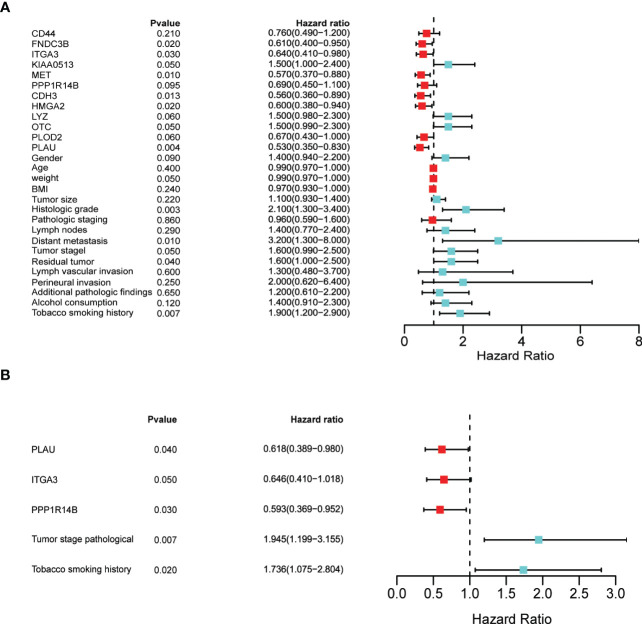
Identification of prognostic factors by univariate and multivariate analyses **(A)** Univariate Cox regression analysis of the following as individual prognostic factors: eight proteins (PLAU, MET, ITGA3, CDH3, FNDC3B, HMGA2, KIAA0513, OTC), weight, histological grade, distant metastasis, tumour stage, residual tumour, and tobacco smoking history. **(B)** Multivariate analysis identified three proteins (PLAU, ITGA3, and PPP1R14B), tumour stage and tobacco smoking history as significant prognostic factors.

To rule out the bias caused by the survival-related clinical parameters in the following analysis, we obtained the clinical dataset from CPTAC-PDAC and screened for the survival-related clinical index by univariate and multivariate cox regression analysis. Univariate Cox regression analyses of the CPTAC-PDAC clinical dataset identified eight proteins (out of the 12) and weight, histological grade, distant metastasis, tumour stage, residual tumour, and tobacco smoking history as individual prognostic factors (**Figure 12A**). Multivariate Cox regression analysis demonstrated that three prognostic proteins (*PLAU*, ITGA3, and PPP1R14B expression) and two clinicopathological factors (tumour stage and tobacco smoking history) were significantly associated with poor survival (**Figure 12B**).

### 3.12 *PLAU* and correlated signatures are associated with the basal subtype of PDAC

**Figure 13:**
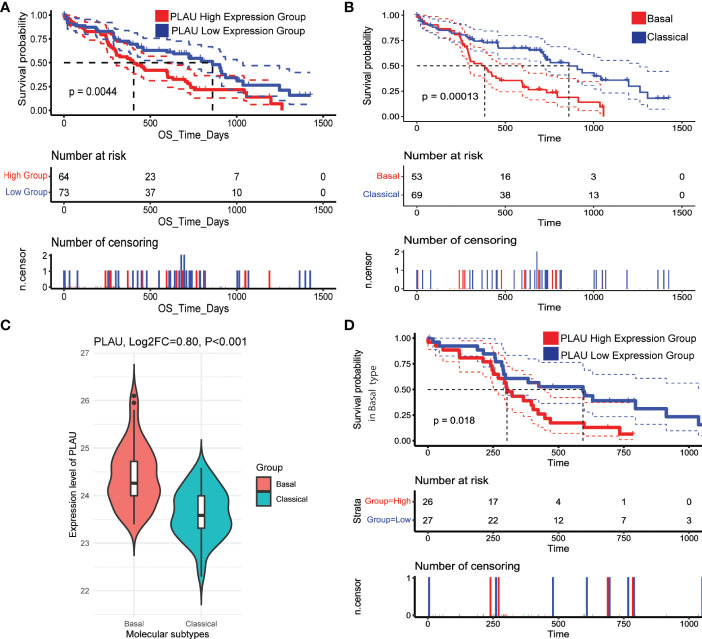
PLAU upregulation is associated with the basal type of PDAC. **(A)** In the CPTAC-PDAC cohort, **(A, B)** Kaplan-Meier survival curves show that increased PLAU protein expression is associated with poor prognosis (log-rank test, P=0044), and the basal subtype of PDAC is associated with worse survival than the classical subtype. **(C)** PLAU protein is significantly upregulated in the basal group than classical subtype (Log2FC=0.80, P<0.001); and **(D)** within the basal subtype, the clinical outcome in the high PLAU expression group is significantly worse than the low PLAU expression group (log-rank test, P=0.018).

Identifying the subtypes of pancreatic cancer could assist with providing the patient with a more accurate prognosis prediction and may also allow precise and effective therapy. Therefore, the association of upregulated *PLAU* protein with survival in patients bearing tumours of PDAC basal and classical subtypes was explored (6). The basal/squamous subtype is characterised by mainly low expression of GATA6 with gene signatures enriched for the inflammation, hypoxia response, metabolic reprogramming and TGF-β signalling, and is also characterised by resistance to chemotherapy and poor outcomes. On the other hand, the classical subtype is characterised by high expression of *GATA6*, KRAS dependency, chemoresponsiveness and a better clinical outcome (4, 72). Using the CPTAC-PDAC cohort, we found that upregulation of *PLAU* protein was associated with poor survival (**Figure 13A**, P=0.0044). Further, a comparison of the survival outcome in basal vs classical clearly shows that the basal group of patients is more at risk of poor prognosis than the classical type (**Figure 13B**). Assessment of *PLAU* protein expression in basal and classical types demonstrated that *PLAU* was significantly more expressed in the basal group than in the classical type (log2FC=0.80, p<0.001, **Figure 13C**). Furthermore, in all three PDAC cohorts (TCGA, ICGC and OICR PDAC cohorts), high *PLAU* gene expression was positively correlated with basal markers including *S100A2* (R=0.48, p=4.57E-10), *FAM83A* (R=0.55, p= 4.38E-13), *IGTA3* (R=0.45, p= 1.14E-08), *KRT5* (R=0.45, p= 6.81E-09), and *C16orf74* (R=0.64, p= 2.48E-18) and negatively correlated with classical molecular subtype markers including *GATA6* (R=-0.57, p= 2.93E-14) *TFF2* (R=-0.42, p= 1.20E-07), *REG4* (R=-0.40, p= 5.98E-07), *LGALS4* (R=-0.39, p= 7.45E-07), and *DDC* (R=-0.44, p= 2.22E-08) (4, 6, 73, 74) (**ST 18**). Of note, survival analysis in the basal group patients demonstrated poor survival outcomes when stratified into the *PLAU* high group compared to the *PLAU* low group (**Figure 13D**, P=0.018). Further survival analysis between *PLAU* high basal versus *PLAU* high classical shows that even though upregulation of *PLAU* is found in both basal and classical group patients, *PLAU* high basal is worse than *PLAU* high classical (**SF7B**, P<0.0001). Consequently, the high and the low in the classical group patients demonstrated no significant association with poor survival (**SF7A**, P=0.9). These results support the concept that upregulation of *PLAU* protein is clinically associated with the poorest survival outcomes in the basal subtype of PDAC.

### 3.13 Effect of uPA - inhibition and Gemcitabine on tumour volume and metastasis *in vivo*

**Figure 14:**
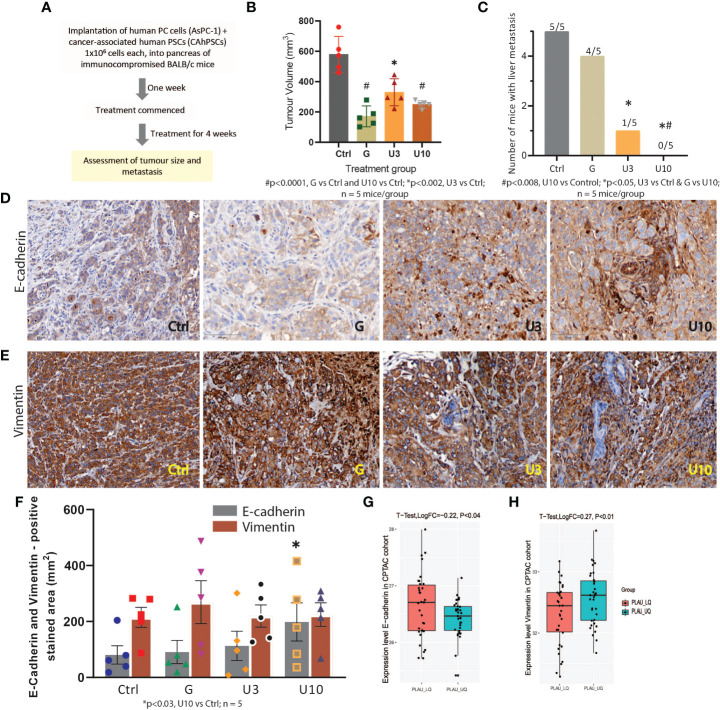
*In vivo* study to assess the effects of PLAU/uPA inhibition and Gemcitabine on tumour progression in an orthotopic model of pancreatic cancer **(A)** Flowchart depicting study design for the orthotopic model. **(B)** Effects of Gemcitabine **(G)**, uPA inhibitor BB230F 3 and 10 mg/kg body weight (U3) (U10), respectively, on endpoint primary tumour volume. Mice bearing orthotopic pancreatic tumours received G, 75 mg/kg body weight twice weekly or U3 or U10 by daily intraperitoneal injections for 28 days. Both gemcitabine and uPA inhibitors significantly reduced tumour volume (n = 5 mice/group). **(C)** uPA inhibition significantly reduced (U3) or completely abolished (U10) liver metastases in mice, while Gemcitabine did not have any effect on metastasis compared to untreated controls. (n = 5 mice/group). **(D, E)** Immunostaining for the mesenchymal marker vimentin and epithelial marker E-Cadherin. Representative photomicrographs depicting staining for E-cadherin and vimentin in mouse pancreas. **(F)** Morphometric analysis shows that while vimentin expression was unchanged by the treatments, E-cadherin expression was significantly increased in U10 compared to controls (n = 5 mice/group). E-cadherin and vimentin (EMT markers), scale bars = 100 μm. **(G, H)** In the CPTAC-PDAC cohort, protein expression analysis of EMT markers indicates that patients in the upper quartile of PLAU expression exhibit low E-cadherin (T-test, LogFC=-0.22 P=0.018) and high vimentin levels (T-test, LogFC=0.27 P<0.01) compared to lower quartile group, suggesting increased EMT in the tumours with upregulated PLAU expression.

Finally, we assessed the effects of uPA inhibition on tumour growth and metastasis using the uPA inhibitor BB230F at 3mg (U3) and 10mg (U10)/kg body weight alongside the standard of care drug gemcitabine in an early intervention orthotopic xenograft mouse model of pancreatic cancer (**Figure 14A**). In this model, we observed that uPA inhibition (with U10) was comparable to Gemcitabine in reducing primary tumour volume at the endpoint. Importantly, uPA inhibition was significantly superior to Gemcitabine in reducing liver metastasis (key site in this model), with U10-treated mice showing no evidence of metastasis (**Figures 14B, C**, **SF9A-B** and **ST20**) in the liver. The absence of liver metastases in all animals treated with U10 was confirmed by histology. Since one of the main mechanisms underlying metastasis is increased EMT of cancer cells, we measured EMT in the model by assessing the ratio of expression of the mesenchymal marker vimentin to the epithelial marker E-Cadherin. An increase in the vimentin: E-cadherin ratio is an indicator of increased EMT. In the orthotopic tumours in this model, we found that while vimentin expression was unchanged, E-cadherin expression was significantly elevated in U10-treated mice compared to the other groups in **Figures 14D-F**, suggesting inhibition of cancer cell EMT by *PLAU* inhibition. We support these observations using the CPTAC-PDAC cohort, whereby patients in the upper quartile of the *PLAU* expression group exhibited a significant decrease in E-cadherin and an increase of vimentin compared to patients in the lower quartile of the *PLAU* expression group (**Figures 14G, H** **)**. Furthermore, immunostaining for the stem cell marker ALDH1A1, which plays a role in recurrence, metastasis, and treatment resistance, demonstrated that U10 significantly decreased ALDH1A1 expression compared to the mice treated with control and Gemcitabine alone (**SF8A, B**), suggesting that the uPA inhibition may inhibit cancer stemness.

## 4 Discussion

Pancreatic ductal adenocarcinoma (PDAC) is an overly aggressive cancer with very high recurrence rates and the poorest prognosis of all solid malignancies. The early and rapid development of metastasis (often seen before the detection of a sizeable pancreatic mass) is the primary driver of the poor clinical outcome of this cancer (75–78).

uPA and its cell surface receptor uPAR play a role in multiple stages of tumorigenesis, especially cancer progression (e.g., ECM degradation and EMT) (7, 24–27, 33–41). Moreover, clinical evidence demonstrates that high *PLAU* mRNA expression is associated with significantly worse clinicopathological characteristics and poor prognosis in PC patients (79, 80). In this study, we have elucidated the key molecular pathways modulated by or associated with *PLAU* upregulation. This will not only enable better prediction of clinical outcomes but importantly may help stratify and identify patients who may best benefit from therapeutic targeting of the uPA.

Using TCGA, CCLE and GEO databases, we have convincingly demonstrated that *PLAU* mRNA levels were significantly upregulated in 44 PDAC cell lines derived from primary or metastatic tumours compared to normal tissues. Importantly, analysis of the TCGA and ICGC PDAC cohorts confirmed the prognostic value of *PLAU* in pancreatic cancer. Validation of this finding at the protein level was obtained by analysis of the CPTAC-PDAC cohort, which demonstrated that high *PLAU* protein expression was significantly correlated with poor survival in PDAC patients.

To help understand the mechanisms mediating *PLAU*-associated poor survival, gene signatures that were commonly positively or negatively correlated with *PLAU* upregulation were identified in the TCGA, ICGC and OICR PDAC-specific cohorts. Analysis of these correlated genes revealed that *PLAU* upregulation was associated with gene signatures mainly encoding transcription factors, cytokines, growth factors, protein kinases and oncogene, which are involved with epithelial-mesenchymal transition, ECM degradation, cell proliferation, hypoxia, angiogenesis, stemness and metastasis. Survival analysis revealed that in the TCGA-PDAC cohort, 6% of positive and 7% of negatively correlated gene signatures were associated with poor survival. The key genes and their functions are summarised in **Table 1**. Of the downregulated genes in colon (81) and ovarian (82) cancer, *PPARGC1A* was reported as a tumour suppressor, and downregulation is associated with poor survival in colon cancer (83). However, the significance of the remaining downregulated genes in PDAC prognosis needs to be explored.

Examination of the protein-protein interaction network revealed that PLAU interacted directly with 31 positively correlated signatures that are active in oncogenesis hypoxia, proliferation, ECM degradation and EMT. On the other hand, PLAU interacted directly with one negatively correlated gene, ANG (angiogenin), the high expression of which is reported to be favourable in pancreatic cancer (84).

Gene set enrichment analysis confirmed that *PLAU* and its positively correlated signatures were involved with pathways that play a role in cancers. In contrast, *PLAU* and its negatively correlated signatures were predominantly related to the downregulation of metabolic pathways. With respect to the former group, 11 main pathways were identified, as depicted in **Figure 5**. Of particular interest are the following: i) the Hedgehog signalling pathway - known to be involved in early pancreatic cancer tumorigenesis (85). A component of this pathway Sonic HH (SHH), is increased more than 40-fold in pancreatic cancer stem cells responsible for tumour recurrence (86, 87). Li et al. showed that hypoxia-induced ROS production increases the expression of *PLAU* and MMP2 in pancreatic cancer cells through the Hh signalling pathway to facilitate invasion and metastasis (88). ii) the metabolic pathway glycosaminoglycan biosynthesis - chondroitin sulfate that facilitates invasiveness of cancer cells by supporting the adhesion of various cells such as fibroblasts or leukocytes in the TME which are the source of growth factors and ECM‐degrading enzymes that enable local migration and dissemination of cancer cells (89, 90). Interestingly, upregulation of components of this pathway, chondroitin and dermatan sulfate, has been reported in pancreatic tumours (91). iii) the Wnt signalling pathway, one of the critical cascades regulating development and stemness in cancer (92). This pathway is known to be critical to the initiation and progression of PDAC (93). iv) the TGF-beta signalling pathway which is most significantly involved in EMT induction in pancreatic cancer cells through activation of ERK/MAPK, PI3K, p38, JNK, RhoA, and other signalling pathways (36–38).

Intriguingly, *PLAU* upregulation and its negatively correlated gene signatures were found to be associated with the downregulation of a large number of metabolic pathways. Such downregulation could be attributed to a severely hypoxic environment in the tumour as a result of pronounced desmoplasia that limits oxygen diffusion (94, 95). Indeed, we found a significant increase in hypoxia in the high expression group of *PLAU* (**Figures 6A, C** and **8E**). Given the central role of PSCs in the production of desmoplasia, it was also of interest that a significant correlation was identified between *PLAU* upregulation and activated PSC abundance (R= 0.41, P=2.754e-07) as well as between *PLAU* upregulation and Hypoxia-inducible factor-1α expression (R= 0.53, P<2.2e-16), a known PSC activation factor.

Moreover, PLAU upregulation is negatively associated with these pathways, suggesting that downregulation of critical metabolic pathways in pancreatic cancer patients may result in worse outcomes. Evidence suggests that metabolic disorders and failure of immunosurveillance to prevent malignancies are key drivers of cancer progression. The tumour immune escape phenomenon can be induced by several factors, including the loss of antigenicity, the loss of immunogenicity, and the immunosuppressive tumour microenvironment (TME), which are orchestrated by nutrient limitation and the build‐up of specific metabolites and signalling molecules (96, 97).

The activation of the uPA/uPAR system has been reported to drives aerobic glycolysis (Warburg effect) in melanoma cell lines even in normoxic conditions, and this activation depends on the α5β1-integrin-mediated uPAR connection with EGFR with the engagement of the PI3K-mTOR-HIFα pathway (98). It has been established that the transcription factor HIF-1α promotes aerobic glycolysis and regulates tumour invasion and metabolism (99). Moreover, in this energy-deprived milieu, *PLAU* upregulation was also found to induce more hypoxia and activate the TGF beta pathway, thereby further increasing tumour immune suppression. Based on the above, it would be reasonable to speculate that uPA may participate in altering and/or downregulating metabolic pathways and in facilitating an immunosuppressive environment, thereby ultimately enhancing tumour progression.

*PLAU* upregulation was also associated with other PSC-derived factors and pathways that are thought to mediate the well-established bidirectional interaction between PSCs and PDAC cells. Activated PSCs markers that were positively correlated with *PLAU* (Pearson correlation test R>0.30, P<0.05), including *CDH11* [Cadherin-11 is elevated in PSCs and is related to PC cells migration (100)), *MME* (or CD10+ PSCs augment the aggressiveness of PDAC (101)], *LGALS1* [Galectin-1 plays role in the development and maintenance of an immunosuppressive microenvironment and promotes PDAC cells metastasis (102–104)], *FERMT2* [progression of pancreatic cancer (105)), *S100A4* (mesenchymal markers increased in activated PSCs (106)], *TGFb1* [TGF-beta signalling in activated PSCs promote ECM accumulation, induced EMT etc. (107, 108)], *POSTN* [promote cancer cell survival, EMT, invasion, and metastasis (109, 110)], *Runx2* [regulate the transcription of extracellular matrix modulators *SPARC* and *MMP1* and impact the tumour microenvironment (111)], *IL-1*[immune suppression (112, 113)], *IL8* (crosstalk with endothelial cells (20)), *PGDF* (proliferation and angiogenesis (20, 114)) and *PLOD2* (creates a permissive microenvironment for migration of cancer cells (115)).

The prominent ECM in PDAC not only supports cancer progression by directly promoting cellular transformation and metastasis but also affects the function of stromal cells to induce angiogenesis and inflammation, thereby resulting in a pro-tumorigenic microenvironment (116, 117). ECM proteins have also been recognised as essential components of the metastatic niche to maintain cancer stem cell properties and enable the outgrowth of metastasis-initiating cells (118–120). Therefore, an analysis of the association of *PLAU* and specific ECM markers and their prognostic significance was also undertaken in this study. 49 ECM gene signatures were found to be correlated with *PLAU*, of which three, namely, secreted factors *S100A16* (cancer-cell–derived), ECM regulator *PLOD2* (PSC-derived) and ECM regulator *TGM2* (cancer and stromal cell-derived) were significantly associated with poor survival in the TCGA-PDAC cohort. However, survival analysis using the CPTAC cohort revealed that only PLOD2 protein upregulation was significantly associated with poor survival (**Figure 11A**, PLOD2).

The immune system is now recognised to play a central role in cancer biology. There have been no studies to date assessing the association between *PLAU* expression and immune signatures in PDAC. This study has shown for the first time that *PLAU* expression correlates closely with immune gene signatures in three PDAC cohorts. In fact, upregulation of *PLAU* was associated with immune inhibitory rather than immune-stimulatory signatures. This concurs with the observed association of *PLAU* with growth factors and cytokines known to promote an immunosuppressive tumour microenvironment.

In view of the positive association discussed above between *PLAU* and its correlated signatures and factors that signify tumour aggressiveness, high and low *PLAU* groups in the TCGA-PDAC cohort were analysed. The results confirmed that tumours of patients with high *PLAU* gene expression also exhibited significantly increased proliferation, EMT, stemness, ECM degradation, hypoxia and immunosuppressive TME. These results suggest that *PLAU* and its correlated signatures induce an aggressive cancer phenotype leading to poor survival.

As outlined above, this study has clearly established that dysregulated *PLAU* and its correlated gene signatures have the potential to confer a poor prognosis for PDAC. However, without knowledge of related changes in the proteome, the usefulness of prognosis prediction based on only gene expression remains a challenge. Proteins are the key functional drivers of cancer biology, providing a link between genotype and phenotype and are common targets of anticancer drugs. Thus it is important to note that, using the CPTAC-PDAC cohort, most of the *PLAU* correlated prognostic gene markers identified in the TCGA-PDAC cohort were also found to be differentially expressed at the protein level. Eleven proteins were associated with poor survival, including upregulated CD44, CDH3, FNDC3B, HMGA2, ITGA3, MET, PPP1R14B, and PLOD2 and downregulated KIAA0513, OTC, and LYZ. We further confirmed their expression level in HPA. Out of 11 ITGA3, MET, FNDC3B, PPP1R14B and KIAA0513, including *PLAU*, were previously reported as individual prognostic markers in the pancreatic cancer TCGA-PAAD cohort (84). However, we have shown these for the first time in PDAC as prognostic markers in our analysis at the transcriptome and proteome levels.

Univariate analysis showed that *PLAU*, CDH3, FNDC3B, HMGA2, ITGA3, MET, KIAA0513, OTC, weight, histological grade, distant metastasis, tumour stage, residual tumour, and smoking are individual prognostic factors for PDAC. Notably, multivariate analysis revealed that *PLAU* protein upregulation in association with ITGA3, and PPP1R14B expression, tumour stage, and smoking history could predict poor overall survival in PDAC. Overexpression of ITGA3 was confirmed in PDAC clinical specimens and associated with poor prognosis (121). Pan-cancer analysis revealed that increased PPP1R14B expression correlated with poor prognosis and increased immune infiltration levels in myeloid-derived suppressor cells (MDSCs), and PPP1R14B could be used as a prognostic biomarker for pan-cancer (122).

The systematic approach used in this study, based on integrated proteotranscriptomics data, supports a major role for the *PLAU* gene and its corresponding protein (uPA) in driving an aggressive metastatic phenotype of PDAC associated with an immunosuppressed TME. The challenge in using this knowledge to develop *PLAU*-targeted treatment is the well-known heterogeneity of this disease. Therefore, accurate patient stratification is essential to ensure optimal outcomes of targeted therapies. To this end, this study also sought to identify whether specific subtypes of PDAC were associated with *PLAU* upregulation. As noted earlier, the commonest classification of PDAC is based on the morphological features of the tumour, with patients being classified as having classical or basal-like subtypes of PDAC (123). Interestingly, this study found a strong correlation between PLAU upregulation and basal type of PDAC while negatively correlated with classical type gene signatures. Pathway analysis further revealed that *PLAU* upregulation was directly associated with vital oncogenic pathways (WNT, WNT beta-Catenin (93, 124, 125) and EMT (TGF beta (126) pathways as well as with hypoxia and ECM-rich stroma, all characteristic of basal PDAC (127–129). Finally, the acquisition of all the malignant phenotypes in the high PLAU group supports the basal type PDAC association with *PLAU*. The association of high *PLAU* with the basal PDAC subtype was also validated at the protein level using the CPTAC-PDAC cohort, as was the correlation of the basal subtype with poor survival (**Figures 13B-D** and **SF7**).

Importantly, we have validated the concept of a key role for *PLAU*/uPA in cancer progression and its potential as a therapeutic target by performing studies in an orthotopic pancreatic tumour model. Our underlying initial strategy for this study was also to compare a non-chemotherapy targeted approach (uPA inhibitor) with a single agent well-tolerated chemotherapy so as to minimise toxic effects while, at the same time, potentially increasing treatment efficacy. This approach has resulted in very encouraging results where uPA inhibition alone significantly reduced tumour growth to a degree similar to Gemcitabine. Crucially, uPA inhibition was significantly superior to Gemcitabine in reducing metastasis, with U10-treated mice showing no evidence of metastasis. The inhibition of metastasis by uPA inhibition is likely mediated by the decrease in EMT and stemness evident in U10-treated mice. Using uPA knock-out cells Fang et al. has convincingly demonstrated that the knockdown *PLAU* in KYSE-30 cells exhibited significantly reduced tumour growth and weight than the control (normal uPA expression) group, while the *PLAU* overexpression group exhibited increased tumour growth and weight compared with the control group (27). *In vitro* studies using pancreatic cancer cell lines have shown that the knockdown of uPA reduces cancer cell migration, invasion and viability (130).Multiple *in vivo* studies have shown that inhibiting uPA with antibodies, uPA-directed prodrugs or radioisotopes and small molecule inhibitors alone or in combination with other drugs can block cancer growth, invasion and metastasis in prostate and breast cancer (131–135). In addition, uPA inhibitors have also demonstrated very encouraging outcomes in clinical trials for the treatment of different types of solid tumours (136, 137), including using Upamostat (WX-671, Mesupron) in advanced pancreatic cancer patients (138, 139).

This study has yielded novel findings regarding *PLAU* and its role in PDAC tumour progression using comprehensive and integrated transcriptomic/proteomic bioinformatic analyses. Moreover, since upregulation of *PLAU* levels is also frequently observed in a number of malignancies and upregulation of *PLAU* is a prognostic marker not only in pancreatic cancer but also in head and neck, endometrial cancer, renal and lung (42), breast (140) and oesophageal cancer (27). In light of the above, it is highly likely that the approach used in our study for pancreatic cancer could be a promising approach for several other cancers. However, the study does have limitations. All clinical cohorts in this study (with small sample size) primarily comprised Caucasians or Africans; therefore, caution must be exercised to extrapolate the findings to patients of other ethnicities. The orthotopic xenograft model of pancreatic cancer used in this study involved using a mixture of human cancer cells and human pancreatic stellate cells that provided strong support for our concept that uPA drives pancreatic cancer progression. However, the mice were necessarily immunodeficient, and as such, the model did not lend itself to characterise any immune infiltration into the tumours accurately. The findings derived from our *in silico* and *in vivo* analyses need to be validated experimentally in more depth, a step currently being pursued in our laboratory. In this regard, we are evaluating the effects of inhibiting uPA in a clinically representative orthotopic mouse model (early and advanced) of PDAC in both immune-deficient and immune-competent (syngeneic KPC model, where a mixture of mouse cancer cells and mouse pancreatic stellate cells is implanted into the KPC mouse pancreas) settings with more numbers of mice. The immune cell landscape in this model closely resembles that of human pancreatic cancer with infiltration of myeloid-derived suppressor cells (MDSC), Treg cells and a few CD8+ cytotoxic T cells (141–143). Future work will also combine inhibition with multiagent chemotherapy to further optimise outcomes or to demonstrate that single-agent chemotherapy + targeted therapy may be preferred to current multiagent strategies in selected patients. In search of treatment alternatives, we also hypothesise that in basal-like tumours, since upregulation of the PLAU group has higher hypoxia scores and higher immunosuppressive tumour signatures (PD-L1 and PD-L2) than the anti-tumour immune signature (CD8+ T cells), which may be predictive of immunotherapy (in combination with uPA and plus-minus chemotherapy) in this chemo resistant.

## 5 Conclusion

For the first time, this study has comprehensively revealed the significance of PLAU in PDAC development, metastasis, and immune suppression and has demonstrated the potential translational importance of inhibiting master regulator PLAU protein in basal type PDAC patients. Thus, it would not be unreasonable to hypothesise that selectively inhibiting PLAU (with and without chemo/immune therapy) in patients with basal PDAC may represent a novel and effective therapeutic approach to improve patient outcomes.

## Data availability statement

Publicly available datasets were analyzed in this study. This data can be found here: https://www.ncbi.nlm.nih.gov/gds), Cancer Cell Line Encyclopedia (CCLE, https://depmap.org/portal/ccle/), International Cancer Genome Consortium (ICGC, https://icgc.org/), the Cancer Genome Atlas (TCGA, http://cancergenome.nih.gov/), the National Cancer Institute’s Clinical Proteomic Tumor Analysis Consortium (CPTAC, https://pdc.cancer.gov/pdc/) and the Ontario Institute for Cancer Research (OICR) PDAC cohort’ gene expression and clinical data are only available upon through a data access agreement with referenced institute.

## Ethics statement

The animal study was reviewed and approved by University of New South Wales Animal Care and Ethics Committee (Approval Number 18/125B) and accomplished under ARRIVE guidelines.

## Author contributions

SH. designed experiments, acquired and analysed data, interpreted results, and wrote and revised the manuscript. MU helped analysed data and interpret bioinformatics results. ZX acquired data for *in vivo* experiments; BB synthesized and prepared formulation for *in vivo* study. CP acquired data for *in vitro* experiments. TP helped to interpret the results. AM acquired data for *in vivo* experiments. MM helped to interpret the bioinformatics results. FN and SG acquired OICR-cohort data. RP helped to interpret results and revised manuscript. JW helped to interpret results and revised manuscript. MR helped to interpret results and revised manuscript. DG helped to interpret results, and revised manuscript and MA conceived, designed the study and experiments, interpreted results, and revised manuscript. All authors contributed to the article and approved the submitted version.

## Glossary

**Table:**

TCGA	The Cancer Genome Atlas
ICGC	International Cancer Genome Consortium
OICR	Ontario Institute for Cancer Research
CPTAC	Clinical Proteomic Tumor Analysis Consortium
CCLE	Cancer CellLine Encyclopedia
GEPIA	Gene Expression Profiling Interactive Analysis
ACC	adrenocortical carcinoma
BLCA	bladder urothelial carcinoma
BRCA	breast invasive carcinoma
CESC	Cervical squamous cell carcinoma and endocervical adenocarcinoma
CHOL	Cholangiocarcinoma
COAD	colon adenocarcinoma
DLBC	lymphoid neoplasm diffuse large B-cell lymphoma
ESCA	esophageal carcinoma
GBM	glioblastoma multiforme
HNSC	head and neck squamous cell carcinoma
KICH	Kidney chromophobe
KIRC	kidney renal clear cell carcinoma
KIRP	kidney renal papillary cell carcinoma
LAML	acute myeloid leukemia
LGG	brain lower grade glioma
LIHC	Liver hepatocellular carcinoma
LUAD	lung adenocarcinoma
LUSC	lung squamous cell carcinoma
MESO	Mesothelioma
OV	ovarian serous cystadenocarcinoma
PAAD	Pancreatic adenocarcinoma
PCPG	Pheochromocytoma and Paraganglioma
PRAD	prostate adenocarcinoma
READ	rectum adenocarcinoma
SARC	Sarcoma
SKCM	Skin cutaneous melanoma
STAD	stomach adenocarcinoma
TGCT	testicular germ cell tumors
THCA	thyroid carcinoma
THYM	thymoma
UCEC	uterine corpus endometrial carcinoma
UCS	uterine carcinosarcoma
UVM	Uveal Melanoma
HEG	High Expression Group
LEG	Low Expression Group
FDR	false discovery rate
HIF1A	Hypoxia inducible factor 1 subunit alpha
HLAs	human leukocyte antigens
IFN	interferon
MDSCs	myeloid-derived suppressor cells
NK	natural killer
OS	overall survival
PD-1	Programmed cell death protein 1
PDL1	programmed death-ligand 1
PFI	progression-free interval
SGSs	stromal gene signatures
ssGSEA	single-sample gene-set enrichment analysis
TILs	tumor-infiltrating lymphocytes
OG	Oncogenes
TF	Transcription factors
CK and GF	Cytokines and Growth factors
TCG	Translocating cancer genes
CDM	Cell Differential markers
PK	Protein Kinase
HP	Homeodomain Protein
TS	Tumour Suppressor
EA	Endothelial
STM	Stemness
HYPOX	Hypoxia
CP	Cell Proliferation
ECMD	ECM degradation
ECM	Extracellular Matrix
EMT	Epithelial mesenchymal transition
MTGs	Metastasis-related genes/Metastasis-promoting genes
Ctrl	Control
G	Gemcitabine
	uPA inhibitor (BB2-30F) at 3mg (U3) and 10 mg (U10)
PSCs	Pancreatic stellate cells
PC	Pancreatic cancer
CAhPSCs	Cancer associated human pancreatic stellate cells
