# Supplementary figures and images for: Metastatic phenotype and immunosuppressive tumour microenvironment in pancreatic ductal adenocarcinoma: Key role of the urokinase plasminogen activator (PLAU)
Source: Front Immunol. 2022 Dec 14;13:1060957. doi: 10.3389/fimmu.2022.1060957 (PMC9794594; doi:10.3389/fimmu.2022.1060957)

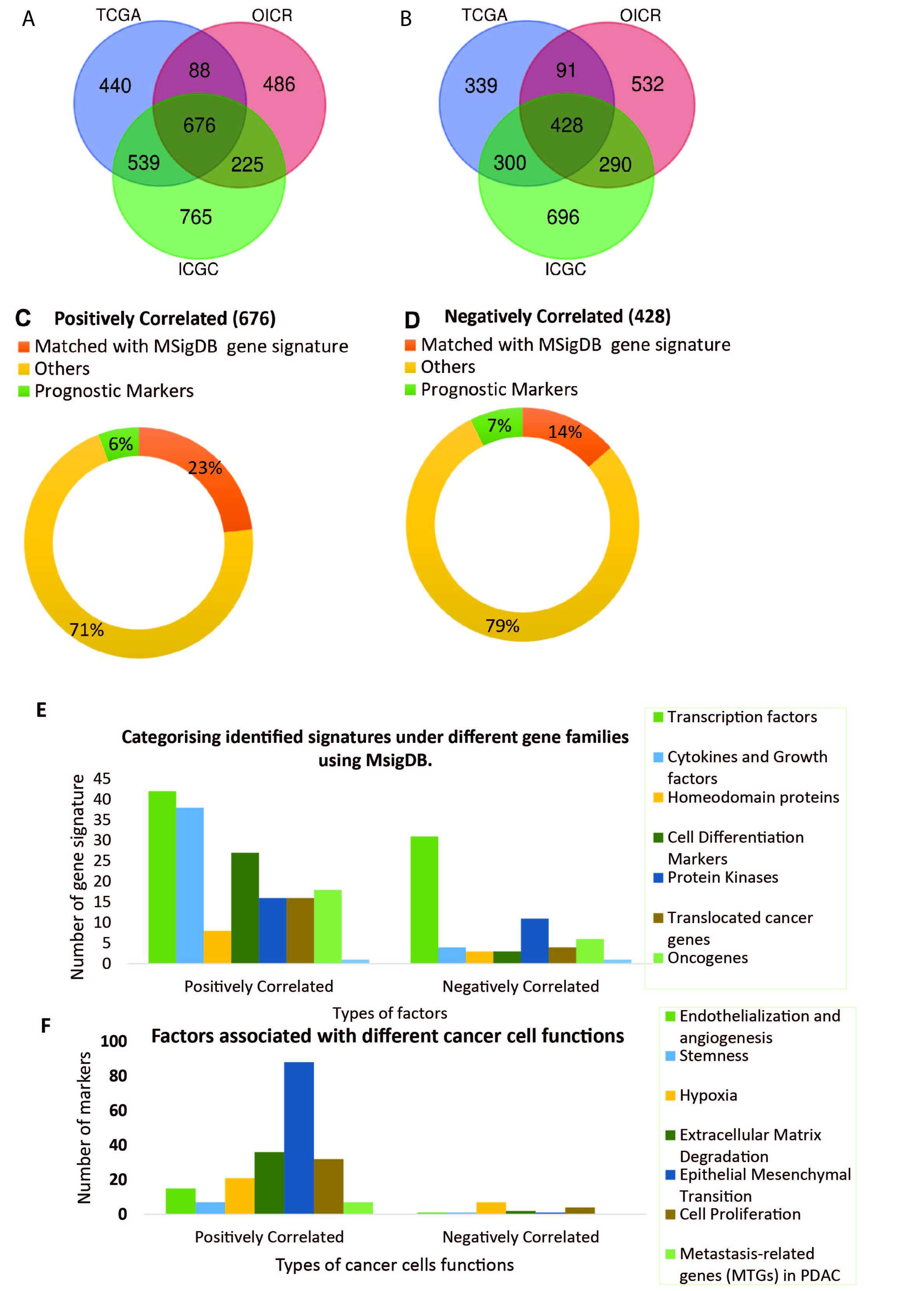

Supplement: Supplementary file 2 [file Image_1.jpeg]

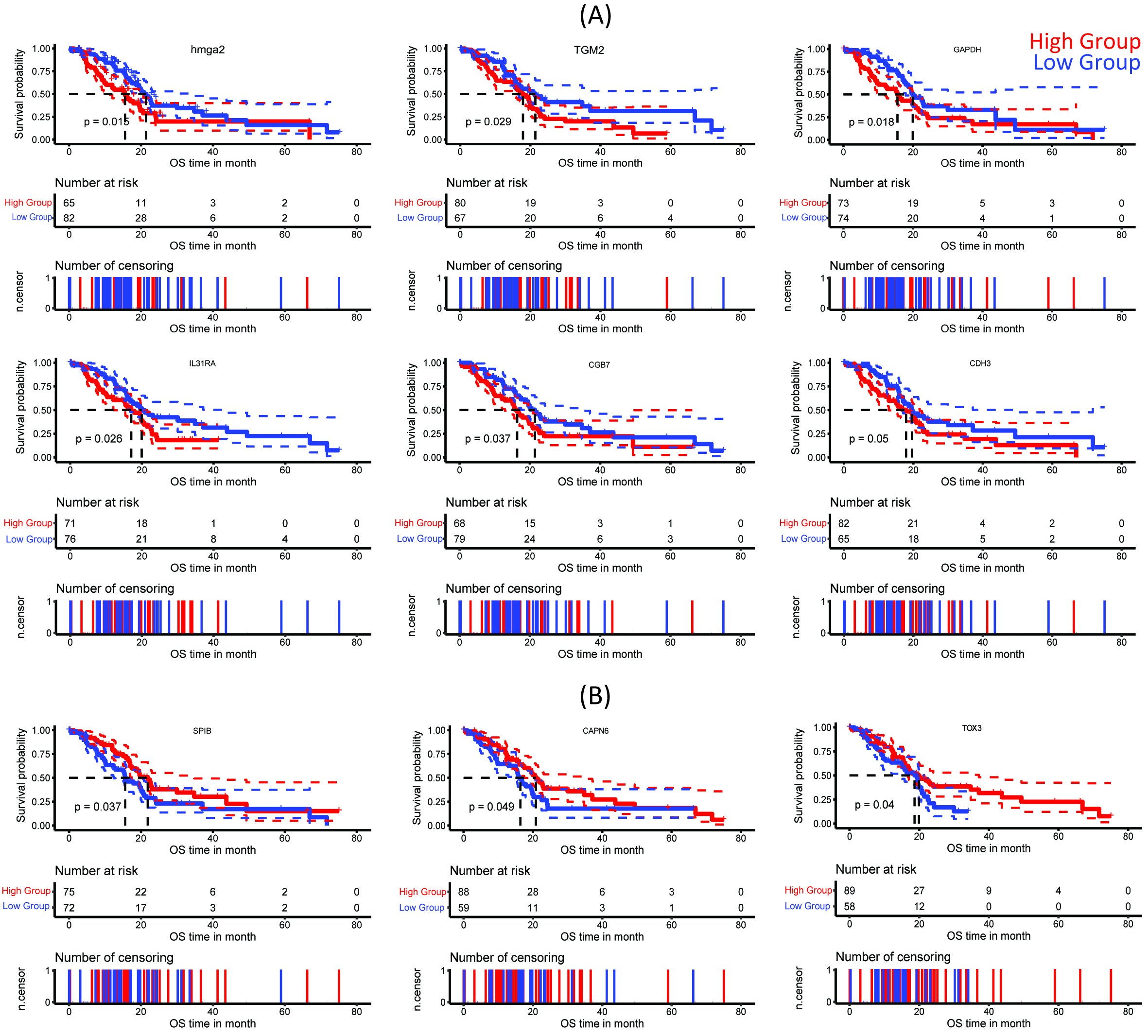

Supplement: Supplementary file 3 [file Image_2.tif]

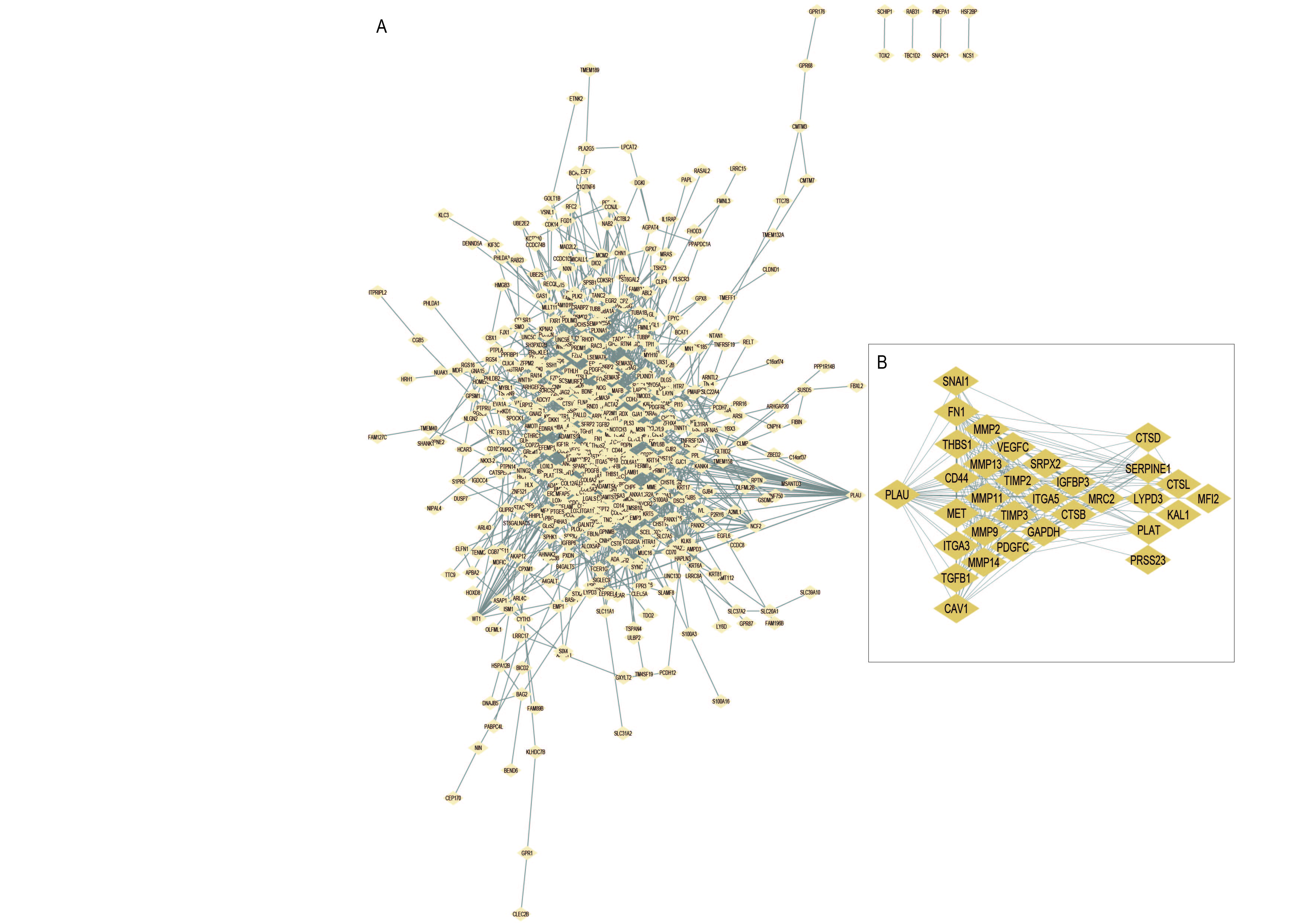

Supplement: Supplementary file 4 [file Image_3.jpeg]

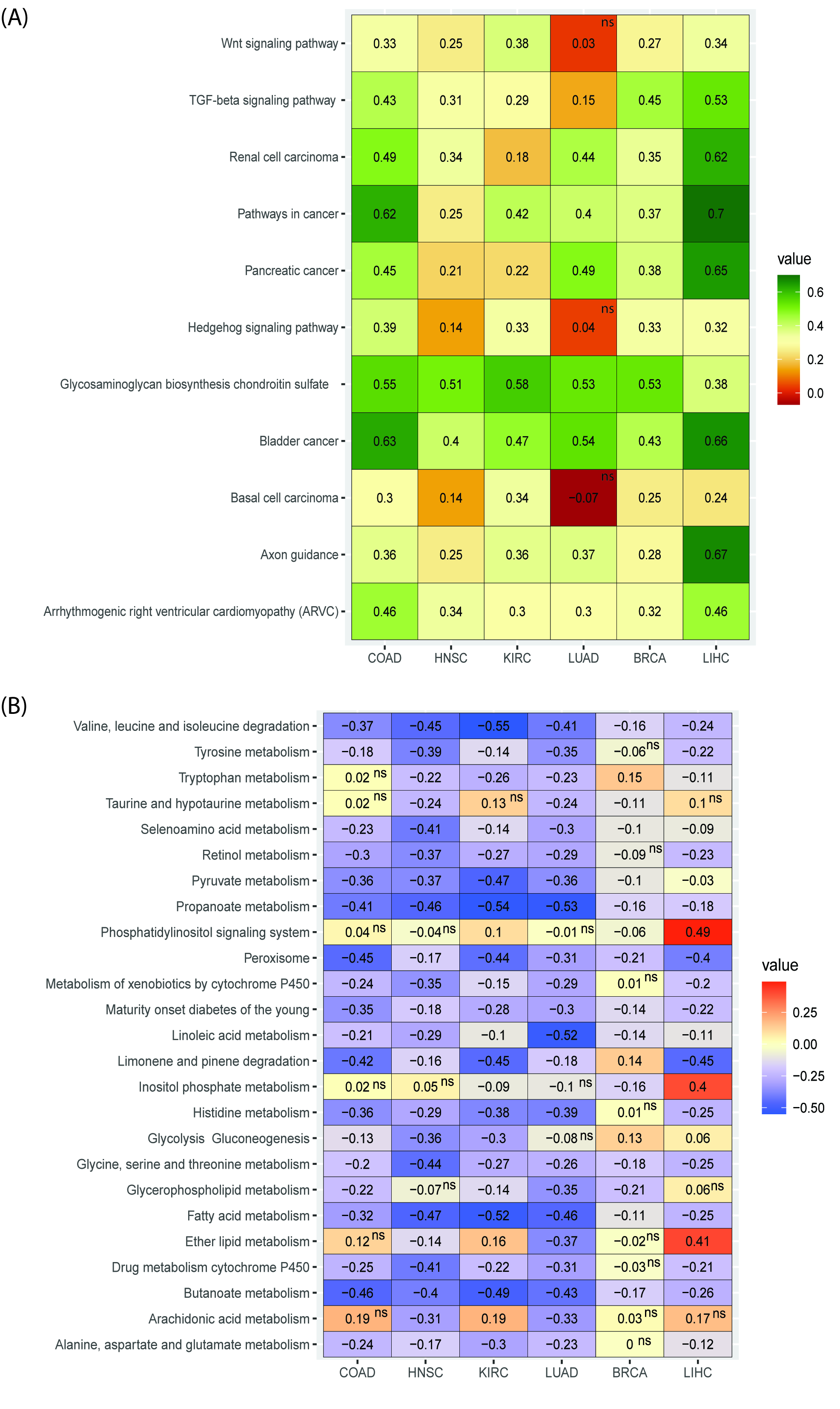

Supplement: Supplementary file 5 [file Image_4.tif]

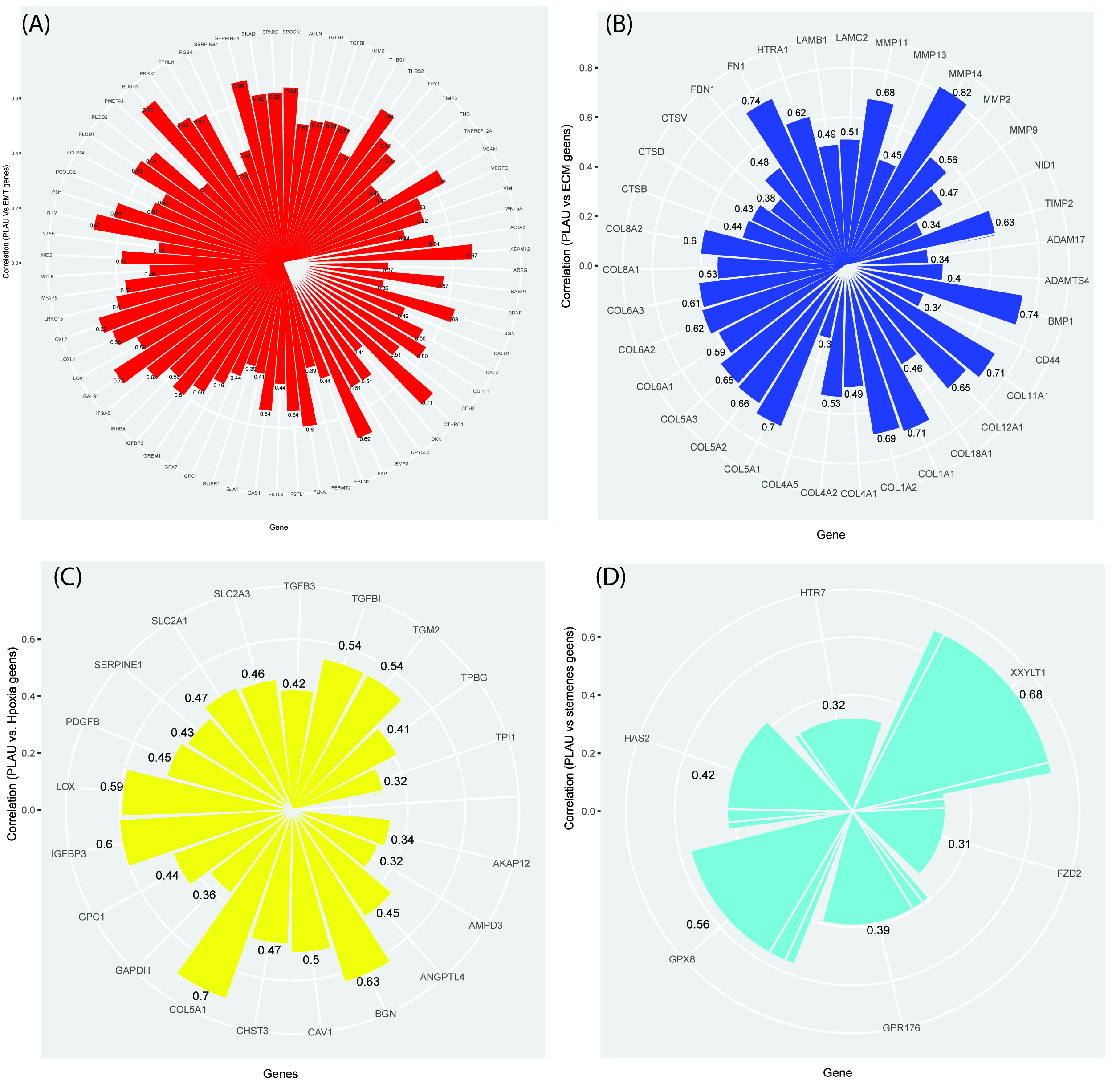

Supplement: Supplementary file 6 [file Image_5.tif]

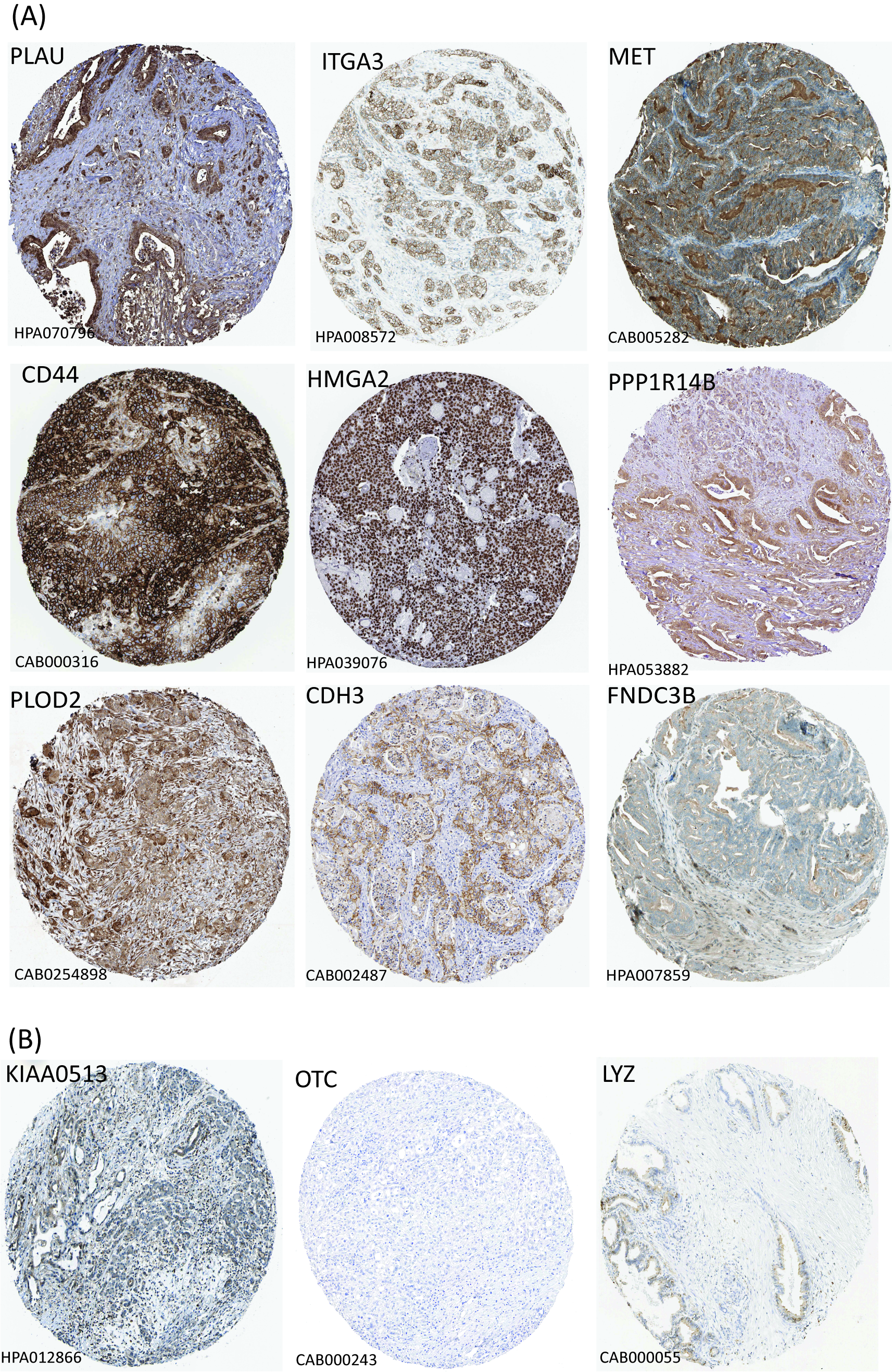

Supplement: Supplementary file 7 [file Image_6.tif]

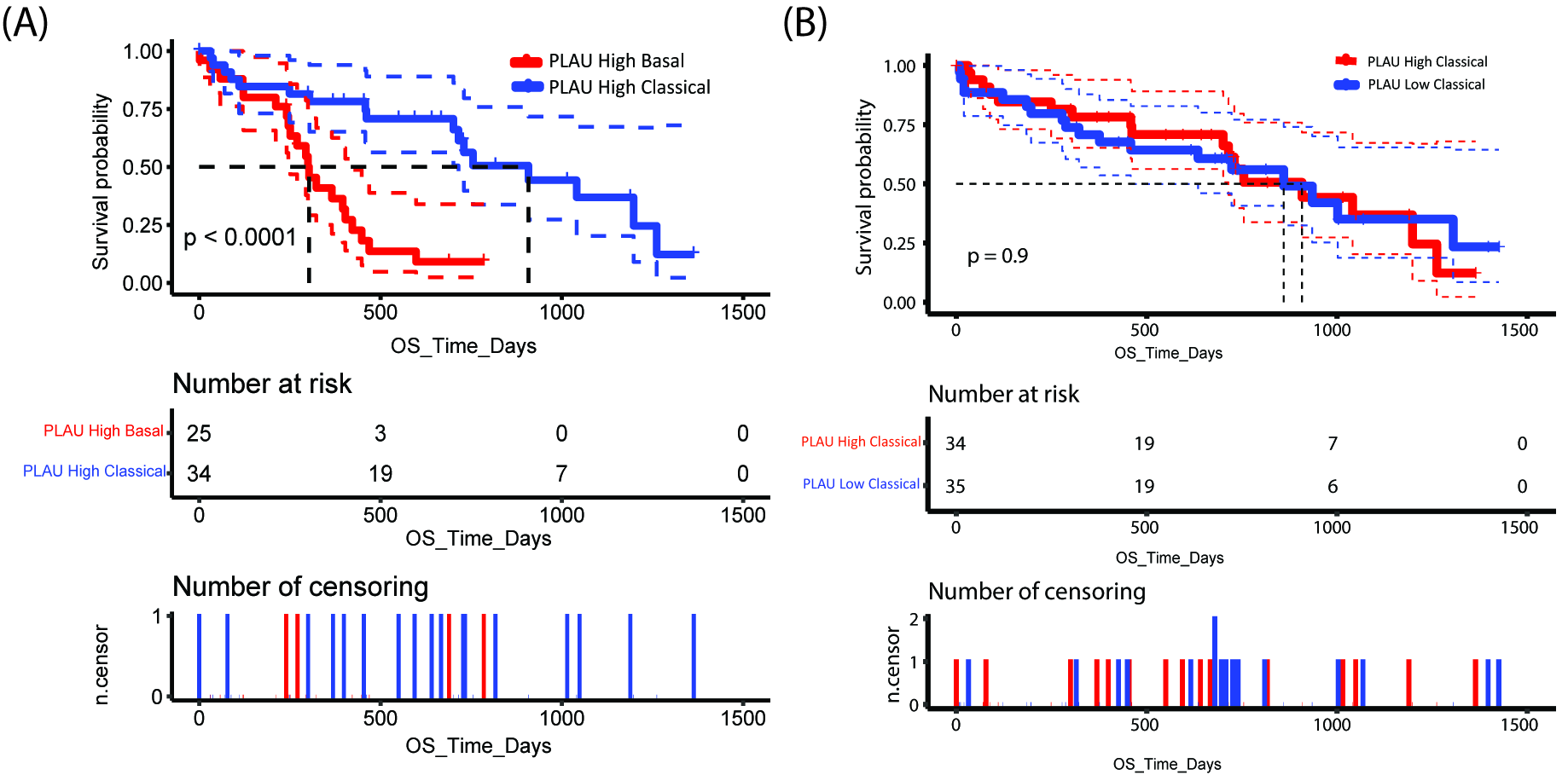

Supplement: Supplementary file 8 [file Image_7.tif]

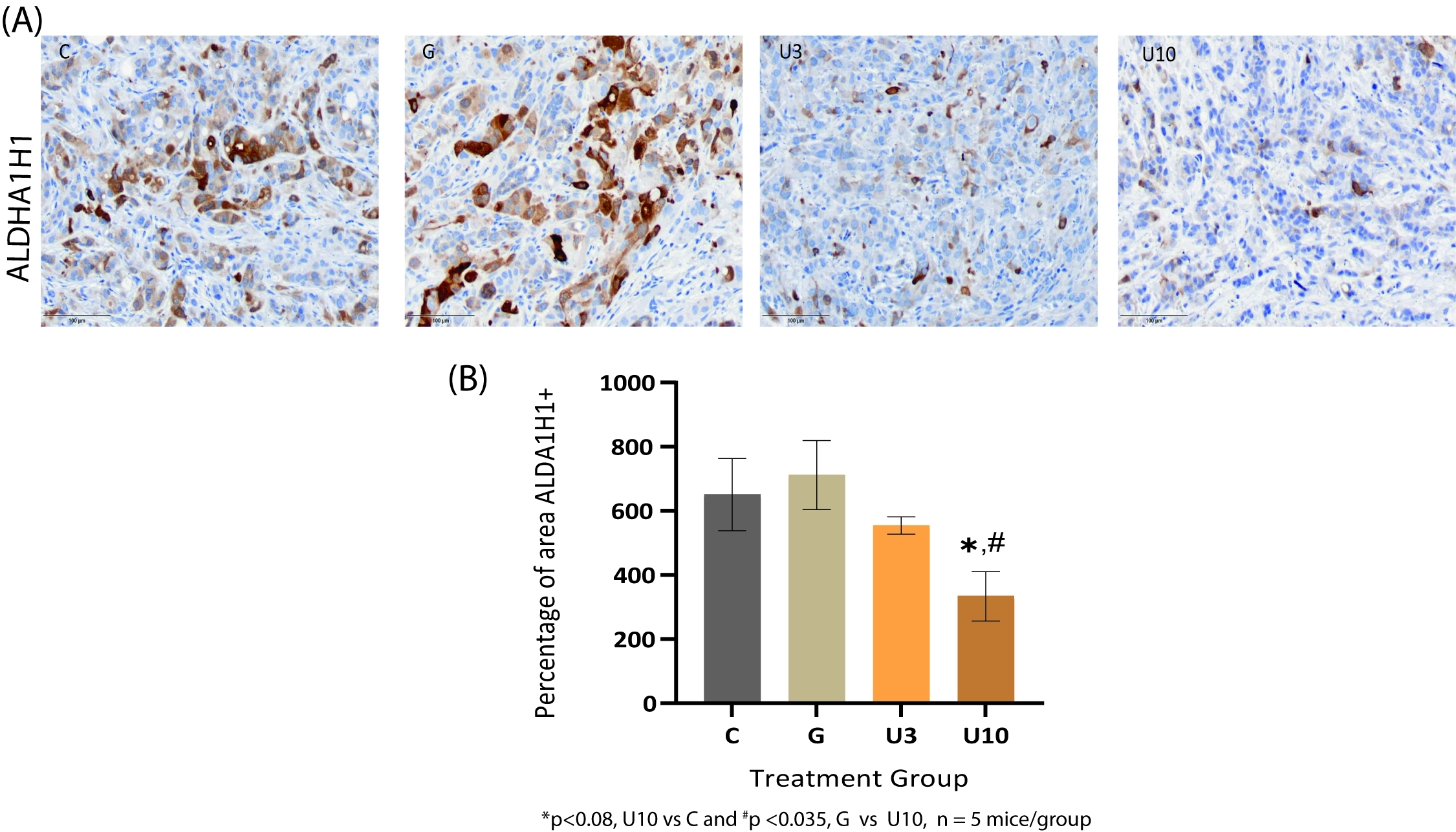

Supplement: Supplementary file 9 [file Image_8.tif]

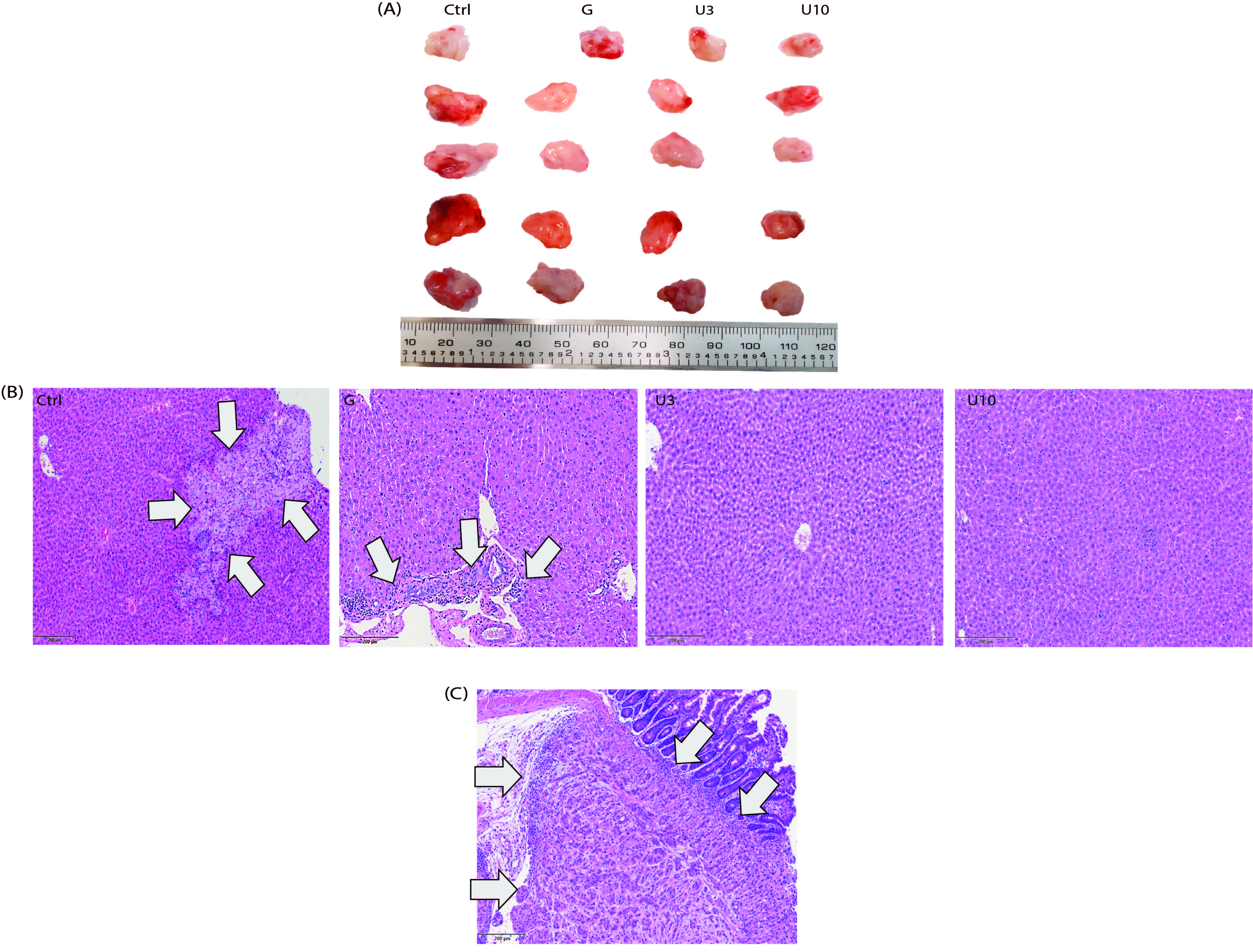

Supplement: Supplementary file 10 [file Image_9.jpeg]
